# Neurosteroids, Microbiota, and Neuroinflammation: Mechanistic Insights and Therapeutic Perspectives

**DOI:** 10.3390/ijms26147023

**Published:** 2025-07-21

**Authors:** Amal Tahri, Elena Niccolai, Amedeo Amedei

**Affiliations:** 1 Department of Experimental and Clinical Medicine, University of Florence, Largo Brambilla 3, 50134 Florence, Italy; amal.tahri@unifi.it; 2 Laboratorio Congiunto MIA-LAB (Microbiome-Immunity Axis Research for a Circular Health), University of Florence, Largo Brambilla 3, 50134 Florence, Italy

**Keywords:** neurosteroids, gut microbiota, central nervous system, gut–brain axis, neuroinflammation, therapeutic perspectives

## Abstract

The gut–brain axis (GBA) represents a complex bidirectional communication network that links the gut microbiota (GM) and the central nervous system (CNS). Recent research has revealed that neurosteroids (NSs) play crucial roles in modulating neuroinflammatory responses and promoting neuroprotection. Meanwhile, GM alterations have been associated with various neuroinflammatory and neurodegenerative conditions, such as multiple sclerosis, Alzheimer’s disease, and amyotrophic lateral sclerosis. This review aims to provide a comprehensive overview of the intricate interactions between NS, GM, and neuroinflammation. We discuss how NS and metabolites can influence neuroinflammatory pathways through immune, metabolic, and neuronal mechanisms. Additionally, we explore how GM modulation can impact neurosteroidogenesis, highlighting potential therapeutic strategies that include probiotics, neuroactive metabolites, and targeted interventions. Understanding these interactions may pave the way for innovative treatment approaches for neuroinflammatory and neurodegenerative diseases, promoting a more integrated view of brain health and disease management.

## 1. Introduction

The central nervous system (CNS) is no longer seen as an isolated organ but as part of a dynamic network that links with peripheral systems, especially the gastrointestinal tract. This connection is known as the gut–brain axis (GBA), a two-way communication system that integrates neural, immune, endocrine, and metabolic signals between the brain and the gut [1,2]. Within this axis, the gut microbiota (GM) and neurosteroids (NSs) have emerged as key modulators of neuroinflammation and neurodegeneration. Recent research increasingly highlights the complex interplay between these systems and their impact on CNS health.

NSs are synthesized in the brain from cholesterol. They rapidly influence neuronal excitability, synaptic function, and neuroplasticity [3,4]. Compounds like allopregnanolone (ALLO) and dehydroepiandrosterone (DHEA) show anti-inflammatory and neuroprotective effects by modulating microglial activity, cytokine release, and oxidative stress [5,6]. Alterations in neurosteroidogenesis is linked to neurodegenerative diseases, such as multiple sclerosis, Alzheimer’s disease, Parkinson’s disease, and amyotrophic lateral sclerosis (ALS) [7,8,9,10].

Similarly, the GM plays a critical role in CNS homeostasis and immune regulation. Dysbiosis, the imbalance in gut microbial communities, contributes to systemic inflammation and disrupts the blood–brain barrier (BBB) integrity, both of which are implicated in neurodegenerative diseases [11,12]. In addition, gut microbes produce metabolites, such as short-chain fatty acids (SCFAs), tryptophan derivatives, and secondary bile acids. These compounds influence microglial maturation, astrocyte function, and peripheral immune cell trafficking to the CNS [2,13,14].

Recent studies have begun to explore how these two systems, NSs and GM, can interact. Some microbial metabolites can regulate enzymes involved in NSs biosynthesis, such as 5α-reductase and 3α-hydroxysteroid dehydrogenase [15,16]. In contrast, NSs influence gut function by modulating immune responses and maintaining epithelial integrity [17,18,19].

Understanding how NSs and GM interact opens new avenues for treating neurodegenerative diseases. Interventions like probiotics, prebiotics, dietary modulation, and neurosteroid supplementation may offer innovative therapeutic strategies.

This review summarizes the current knowledge on molecular mechanisms connecting neurosteroids, gut microbiota, and neuroinflammation. We first outline NSs biosynthesis and function, then examine the GM’s role in neuroinflammation. Finally, we explore how NSs and GM interact and discuss the potential therapeutic applications targeting this axis.

## 2. Neurosteroids

### 2.1. Definition and Classification

NSs are a specific class of steroids produced natively in the CNS and independently of the endocrine gland [20]. Acting as endogenous neuromodulators, NSs have a high impact on various neurophysiological functions, including neurotransmission, neuronal development, and myelination [20]. Unlike classical steroid hormones, which act primarily through nuclear receptors to induce genomic effects, NSs can interact with a wider range of targets, including nuclear, intracellular, and membrane receptors [20]. For example, they can bind to G protein-coupled receptors, modulating neuropeptide signalling, or ligand-gated ion channels, producing rapid non-genomic effects such as changes in neuronal and glial excitability [21]. These non-genomic effects are mediated by interactions with receptors such as type A gamma-aminobutyric acid (GABA_A_), L and T-type calcium channels, N-methyl-D-aspartate (NMDA), and sigma-1 [22,23,24,25].

Endogenous NSs are classified mainly into three categories: pregnane, androstane, and sulfated [22] (Table 1).

The first class is called ‘Pregnane NSs’ and includes two key neuroactive compounds: ALLO and allotetrahydrodeoxycorticosterone (THDOC) [26]. In the nervous system, progesterone is synthesized by both glial cells and neurons through the action of enzymes that are widely distributed in the brain [26]. During pregnancy, the levels of progesterone-derived NSs increase significantly but drop sharply after delivery [26]. Given the anxiolytic properties of neuroactive steroids and the observation that their withdrawal is associated with increased anxiety-like behaviour, it has been proposed that NSs fluctuation may contribute to the onset of postpartum depression [26]. In particular, ALLO is one of the most powerful isomers within the pregnane steroids family due to its strong modulatory effects on neural activity [16]. Pregnanolone (PREG) is the 5β epimer of allopregnanolone and both exhibit similar effects [16]. In contrast, the 3β epimer of allopregnanolone, known as isoallopregnanolone (ISOALLO), antagonizes the effects of its 3α counterpart and functions as a negative allosteric modulator at the GABA_A_ receptor [16]. Another isomer, called epipregnanolone, is also considered a negative allosteric modulator on GABA_A_ receptors [16]. Both ALLO and PREG can impact neuronal activity by improving GABAergic neurotransmission [27]. This modulation results in an increase in inhibitory signalling within the brain [27]. Notably, ALLO has been shown to decrease the response to endocrine stress when administered in advance, due to the inhibition of the corticotropin-releasing hormone (CRH) neurons through GABA-mediated mechanisms [27].

The second class, known as ‘Androstane NS’, comprises Androstanediol and Etiocholanone. Androsterone, a metabolite of DHEA, effectively modulates GABA_A_ receptors. As a neurosteroid, DHEA exerts beneficial effects in reducing stress and promoting resilience in humans [28]. It has been shown to attenuate emotional reactivity by controlling negative emotions [29]. Elevated serum DHEA levels increase the activation of the anterior rostral cingulate cortex, which plays a role in the processes of cognitive regulation and the inhibition of negative emotional responses [29]. These characteristics support the classification of DHEA as an anxiolytic agent, primarily through the modulatory action on GABA_A_ receptors [29].

The third class is “Sulfated NSs”, which include pregnenolone sulphate (PS) and dehydroepiandrosterone sulphate (DHEAS). These sulfated NSs, together with DHEA, have demonstrated antidepressant effects in animals and humans [30].

Despite extensive evidence supporting the modulatory effects of DHEA on various membrane receptors, including the GABA_A_, NMDA, and sigma 1 receptors, the precise mechanism underlying its action in the nervous system has not yet been fully elucidated [31].

**Figure 1:**
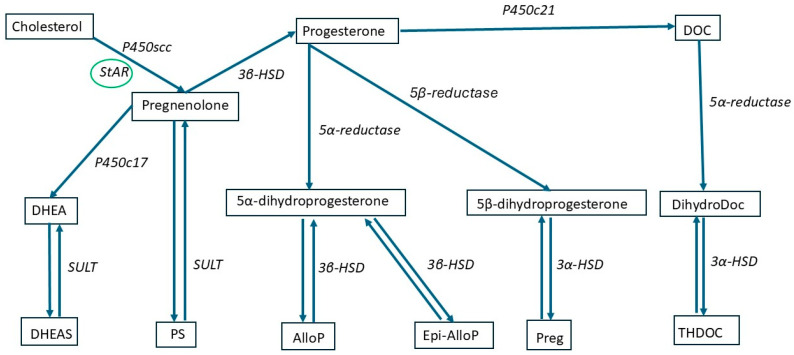
Schematic representation of neurosteroidogenesis pathways. Cholesterol serves as the precursor for neurosteroid synthesis, transported into mitochondria by the Steroidogenic Acute Regulatory Protein (StAR). Conversion to pregnenolone occurs via the P450scc enzyme. Pregnenolone is further metabolized to progesterone by 3β-HSD or to dehydroepiandrosterone (DHEA) via P450c17. Sulfation of pregnenolone and DHEA yields PS and DHEAS, respectively. Progesterone undergoes enzymatic conversions involving 5α-reductase and 5β-reductase to produce 5α-dihydroprogesterone and 5β-dihydroprogesterone, which are further metabolized by 3α-HSD and 3β-HSD into neuroactive steroids such as allopregnanolone (AlloP), epiallopregnanolone (Epi-AlloP), pregnanolone (Preg), and tetrahydrodeoxycorticosterone (THDOC). Progesterone can also be converted to DOC by P450c21, contributing to the pool of neurosteroids via dihydroDOC and THDOC formation. These neurosteroids modulate neuronal excitability and have significant roles in neuroprotection, mood regulation, and stress responses. P450scc: P450 side-chain cleavage, StAR: steroidogenic acute regulatory protein, P450c17: Cytochrome P450c17, P450c21: 21-hydroxylase, 3α-HSD: 3α hydroxysteroid dehydrogenase, 3β-HSD: 3β hydroxysteroid dehydrogenase, SULT: sulfotransferase, DHEA: dehydroepiandrosterone, DHEAS: dehydroepiandrosterone sulphate, PS: pregnenolone sulphate, AlloP: allopregnanolone, Epi-AlloP: epi-allopregnanolone, Preg: pregnanolone, DOC: deoxycorticosterone, THDOC: tetrahydrodeoxycorticosterone.

NSs are produced from a ubiquitous cholesterol precursor [32]. Human steroidogenic cells obtain cholesterol either through the receptor-mediated endocytosis of low-density lipoproteins (LDLs) or by synthesizing it de novo [32]. Generally, most of the cholesterol used for neurosteroidogenesis is derived from LDLs. LDLs are handled by lysosomal acid lipase (LAL) to later generate cholesterol to serve in neurosteroidogenesis [33]. However, cells can use three acetyl CoA molecules to make cholesterol [34]. The translocation of cholesterol through the mitochondrial membrane is considered a rate-limiting step in neurosteroidogenesis and is controlled by two proteins: the ‘steroidogenic acute regulatory protein (StAR)’ and the translocator protein (TSPO) [26,35,36,37,38,39,40,41]. A schematic representation of neurosteroidogenesis pathways is reported in Figure 1.

### 2.2. NSs Mechanisms of Action

The NSs synthesized in the CNS fast regulate neuronal excitability [42]. They can also be classified based on their receptor activity. In fact, inhibitory NSs act as positive allosteric modulators of the GABA_A_ receptor (Table 2). They influence neurotransmission and exert various effects including anxiolytic, stress-reducing, anti-depressant, pro-social, rewarding, pro-sexual, anti-aggressive, pro-sleep, sedative, cognitive and memory improving, as well as anesthetic, analgesic, anti-convulsant, neurogenic, and neuroprotective actions [43]. For instance, 5β-dihydroprogesterone, the progesterone metabolite allopregnanolone (3α,5α-tetrahydroprogesterone or 3a5aTHP), and androstane 3α-androstanediol are well-known representatives of this class [44,45]. On the other hand, excitatory NSs stimulate the nervous system by modulating the GABA_A_, NMDA, and sigma-1 receptors. They are capable of causing cognitive and memory-enhancing, anxiogenic, antidepressant, convulsant, neurogenic, and neuroprotective effects [46]. Excitatory NSs affect neurotransmission by acting as negative allosteric modulators of the GABA_A_ receptor, weak positive allosteric modulators of the NMDA receptor, and agonists of the sigma-1 receptor. By interacting with these receptors or ion channels, NSs regulate brain excitability based on their specific type [46] (Table 2).

NSs containing PS and DHEAS act as agents of the sigma-1 receptor, whereas progesterone-derived NSs function as antagonists. However, these NSs can also modify the GABA_A_ and NMDA receptors. The latter receptors are ion channels that are unlocked when a molecule mimicking calcium links with them. They perform crucial functions such as synaptic plasticity, synaptogenesis, learning, and memory [47] (Table 2).

Although NSs can bind to microtubule-associated protein 2 and promote tubulin polymerisation in cultured neurons, their primary actions occur through the allosteric modulation of neurotransmitter receptors, mainly the GABA_A_ receptor and its central benzodiazepine receptor (CBR) complex [48]. NSs interact with a wide range of GABA_A_ receptor subtypes but show a preference for extrasynaptic receptors containing the delta subunit [49]. Compared to traditional GABA receptor modulators such as benzodiazepines and barbiturates, NSs are considered the most potent [50]. In terms of potency, NSs are equivalent to benzodiazepines and more powerful than barbiturates. However, in efficiency, they are as effective as barbiturates and significantly more effective than benzodiazepines [50]. Most NSs enhance GABA_A_ receptor responses at low GABA concentrations, though specific sulfated NSs can inhibit receptor activity at all GABA levels [51]. These opposing effects arise from distinct binding sites on the receptor [51]. At low GABA concentrations (≤1 μM), NS-mediated potentiation dominates, enhancing neuronal excitability [52]. At higher GABA levels, NSs can directly open GABA chloride channels, although the link between this direct gating and the overall cellular or behavioural effects of NSs remains unclear [52]. NMDA receptors are heterotetramers consisting of two GluN1 subunits and two additional subunits, formed of GluN2 or GluN3 [47]. These receptors have two different agonist-linking sites: one in the GluN1/GluN3 subunits known as the glycine/D-serine binding site and another in the GluN2 subunits called the glutamate binding site [53]. The NMDA receptor has special characteristics that need to be activated. It requires binding to the glutamate agonist and the co-agonist glycine or D-serine [53]. In addition, it requires the liberation of Mg^2+^, stopping the influx of potassium, sodium, and calcium ions, which are generated by an important positive charge of the inner membrane [53]. There are two categories of NMDA receptor antagonists. The first is a competitive antagonist, such as D-2-amino-5-phosphonopentanoate, that links to the glutamate binding site of the NMDA receptor as an alternative to glutamate, avoiding the activity of the NMDA receptor [53]. The second are non-competitive antagonists, better known as ‘open channel blockers’ [54]. Following the activation of the NMDA receptors, Mg^2+^ is removed, and sodium ions can move into the inner membrane, thereby generating a positive charge [55]. Ketamine and phencyclidine, two open-channel blockers, attend GluN2 subunits and stop them, thus blocking the NMDA receptor current. Sulfated-NSs, DHEAS, and PS, have been reported to be powerful allosteric agents in the NMDA receptor complex [55]. Generally, higher micromolar concentrations in DHEAS and PS are needed to achieve action on NMDA-mediated receptor currents [55]. PS can promote the NMDA-mediated response when assessed by electrophysiological records, instead of the assessment of the NMDA-induced rise in intracellular Ca^2+^ when culturing neurons. PS suppresses glycine, GABA, and non-NMDA reactions [55].

The sigma-1 receptor is a chaperone protein located in the mitochondrion-associated endoplasmic reticulum (ER) membrane (MAM) that regulates inositol 1,4,5-triphosphate (IP3) receptors and controls calcium signalling between the ER and mitochondria [25]. Crystallographic studies reveal that the sigma-1 receptor has one domain embedded in the membrane and a C-terminal domain exposed to the cytoplasm. The C-terminal region contains ligand-binding sites and two steroid-binding domains, SBDL1 and SBDL2 [25]. Moreover, the sigma-1 receptor forms a complex with the binding immunoglobulin protein (BiP) [25]. This interaction is disrupted by endogenous neurosteroid ligands, which in turn modulate calcium ion channels [25].

NSs synthesis and activity are likely to be largely impacted by neurotransmitters. This interaction between NSs and neurotransmitters can influence cognition, mood, and behaviour [48]. One key example is GABA, the brain’s primary inhibitory neurotransmitter, which is also produced by the GM. Alterations in the glutamate/GABA circuits are interrelated with the development of several neuropsychiatric disorders, including schizophrenia, autism, and depression [56]. For instance, studies show that specific probiotic strains, such as *Lactiplantibacillus plantarum* and *Levilactobacillus brevis*, can produce GABA [57]. Additionally, certain bacteria, including *Lactobacillus rhamnosus*, modulated GABA receptor expression in mice, reducing anxiety and depression-like behaviours [56].

### 2.3. Pharmacological Properties of NSs

Through their actions on GABA_A_ receptors, NSs can perform a multitude of psychopharmacological actions including anti-depressant, anxiolytic, sedative, anesthetic, anti-convulsant, amnesic, and analgesic effects [58,59,60]. Furthermore, studies carried out on rodents supported that NSs enhance sexual behaviour in women [61], have rewarding properties [62,63], and can affect cocaine or ethanol consumption [64,65,66]. Literally, the acute administration of many addiction drugs such as morphine, nicotine, alcohol, Δ9-tetrahydrocannabinol, and γ-hydroxy-butyric acid, increase the brain and plasma concentrations of NSs, namely 3α,5α-THP and/or its precursor pregnenolone and progesterone, and this can be responsible for their rewarding effects [67,68,69,70,71,72].

In addition to these psychopharmacological actions, NSs have several neuroprotective effects. The mechanism of its neuroprotective action relies on its anti-inflammatory and antioxidative properties. Indeed, to defend the CNS against damage, pregnenolone and DHEA, the most prevalent NSs in the CNS, control the equilibrium between excitation and inhibition in the brain by precisely regulating the GABAergic and glutamatergic ones [73]. These NSs have been reported to favour neuronal survival by decreasing oxidative stress and inflammation and allowing the growth and differentiation of neurons and glial cells, thus promoting brain development and plasticity [74]. pregnenolone and DHEA are involved in the myelination mechanism, which is crucial for nerve impulse transmission [75]. Pregnenolone and DHEA can prevent glutamate and staurosporine-induced cortical neuronal degeneration at physiological concentrations, thereby acting as natural neuroprotective agents [76].

In fact, DHEA is well known for its implication in improving neurogenesis, neurocognitive functions, and neuron survival [77,78]. It shows potent neuroprotective activity in addition to anti-inflammatory and antioxidant properties that lead to positive effects in animal models [79]. For example, DHEA manifested neuroprotective neurogenic effects in both in vitro and in vivo models [80].

Progesterone is an additional NSs compound showing neuroprotective activity in glial cells and hippocampal neurons in vitro [81]. Many preclinical studies validated its anti-inflammatory and neuroprotective effects on neuronal cells in the cranial traumatic brain and after cerebral injuries [82,83]. Its neuroprotective effects have also been confirmed in Wobbler mice models recognized for spontaneous spinal motor neurone degeneration [84,85,86]. Furthermore, progesterone treatment has been suggested to solve neurological disorders such as ALS, peripheral nerve injury, TBI, and cerebral ischemia [87].

Finally, ALLO is a NS that represents a positive allosteric modulator for GABA_A_ receptors, which increases inhibitory neurotransmission [88]. Preclinical studies evaluated its ability and synthetic analogues in the treatment of several neurodegenerative diseases and confirmed their neuroprotective effects [89].

In general, NSs can have psychopharmacological, neurotrophic, and neuroprotective effects in experimental models [6,7,84,90,91,92,93,94].

## 3. NSs and Neuroinflammation

Maintaining CNS homeostasis is based on the equilibrium of innate immunity where the principal actors are glial cells consisting of microglia, astrocytes, and oligodendrocytes [95]. The intensity of the neuroinflammatory response depends on the duration, context, and level of the primary offence [96]. NSs are produced near neurons and glial cells that confer them a prompt action on the nervous system in specific situations. NSs are considered potential therapeutic agents for neuroinflammatory and systemic inflammatory disorders by modulating immune responses within the brain [26]. NSs involve specific receptors and impact inflammation via different complementary mechanisms, including barrier protection, cellular signalling, and oxidative stress regulation. Throughout inflammation, microglia and astrocytes trigger and secrete cytokines that lead to potentiation or subsiding. ALLO and DHEA inhibit the pro-inflammatory cytokines’ liberation, favouring the activity of anti-inflammatory mediators. ALLO decreases microglial activation by suppressing NF-κB and the NOD-, LRR-, and pyrin domain-containing protein 3 (NLRP3) inflammasome pathways. PS manages GABAergic and glutamatergic signalling contributing to the decrease in astrocyte-mediated inflammation. PROG and DHEA further limit inflammation related to oxidative stress by increasing superoxide dismutase (SOD) and glutathione (GSH) levels and acting as reactive oxygen species (ROS) scavengers. Additionally, oestradiol and progesterone help reducing leukocyte infiltration and cytokine entry into the CNS by strengthening tight junctions within the BBB [26]. Moreover, progesterone exerts anti-inflammatory effects on microglia by decreasing the expression of pro-inflammatory genes such as TNF, IL-6, MHCII, iNOS, and COX2. It also suppresses inflammasome activation while promoting the expression of reparative and anti-inflammatory markers such as TREM2, TGF-β, and CD206. These actions are mediated by the activation of membrane progesterone receptors (mPR) and progesterone membrane receptors (PGMRC) 1 and 2 [97]. Furthermore, progesterone therapy decreases the NLRP3 inflammasome, either at the gene or protein level, in addition to its derived product, namely IL-18 [98]. progesterone suppresses NF-κB and MAPK activation in microglia and subsequently inhibits pro-inflammatory mediators [99]. Similarly, estrogens and especially estradiol (E2) activate estrogen receptor α (ERα) as well as the G protein-coupled estrogen receptor (GPR30), thus exerting powerful anti-inflammatory effects. Generally, E2 can mobilize calcium, induce cAMP signalling, and activate ERK1/2 [100]. E2 has been reported to decrease the expression of iNOS, IL-6, and TNF-α expression and NLRP3 in stimulated microglial cells that express the different estrogen receptors [101,102,103]. Furthermore, DHEA induces anti-inflammatory effects by activating the TrkA receptor that suppresses IL-6, iNOS, and TNF-α gene expression after the AKT/CREB signalling cascade [104].

Chronic neuroinflammation can disrupt NSs synthesis and contribute to the development of neurodegenerative diseases such as Parkinson’s disease, Alzheimer’s disease, multiple sclerosis, and ALS [26].

## 4. Gut Microbiota and Neuroinflammation

The gut microbiota, a complex ecosystem that involves a large collection of microorganisms that count more than 10^14^ different microorganisms living in the gastrointestinal tract (GI), has historically been considered a “forgotten organ” of the human body [105,106,107]. It can modulate brain morphology and function by impacting the CNS’s function and behaviour through the gut–brain axis [108,109,110]. For example, germ-free animals (GF) develop brain anomalies [111,112,113,114,115]. Furthermore, the administration of specific microbial strains to animals has been shown to induce behavioural changes [56,116,117,118]. Notably, even the introduction of a single microbial strain has demonstrated the ability to protect against certain systemic immune alterations and stress-related behaviours [119].

Many studies have established a connection between depressive disorders and alterations in specific gut microbiota communities, especially within the *Proteobacteria*, *Firmicutes*, *Actinobacteria,* and *Bacteroidetes* phyla [120,121,122]. Regarding the *Firmicutes* phylum, significant changes in the abundance of *Lachnospiraceae* have been observed in mouse models of depression in mice, showing a strong correlation with inflammatory markers [123]. These findings further support the interplay between GM, stress response, and inflammation in the development of depressive disorders [124]. Microbial metabolites are the main mediators in the relationship between the host and GM [122]. The analysis of fecal metabolites offers a non-invasive approach to unveil the gut–brain axis and gain insights into the mechanisms underlying metabolic and neuropsychiatric disorders [125,126,127,128,129,130,131]. For instance, in patients with ulcerative colitis experiencing depression/anxiety, Yuan et al. reported that reduced microbial diversity was associated with metabolic disturbances and gut dysbiosis [129].

The most relevant categories of GM-derived metabolites include serum bile acid (SBA) metabolites/derivatives, serum amino acids (SAA), and serum short-chain fatty acid (SCFA) metabolites/conjugates [132]. The GM plays a vital role in the metabolism of amino acid metabolism, producing metabolites that can influence the synthesis of various neurotransmitters. Notably, more than 90% of the body’s serotonin, a molecule functioning as a neurotransmitter, hormone, and mitogen, is produced in the gut by enterochromaffin cells, using the tryptophan hydroxylase enzyme [110]. Serotonin acts on adjacent epithelial cells, the enteric nervous system, and immune cells through specific receptors, promoting intestinal motility and peristalsis [110]. Certain gut bacteria can modulate the production of metabolites that enhance serotonin biosynthesis in the colon, probably upregulating tryptophan hydroxylase expression [133]. Experimental studies suggest that GF animals exhibit reduced circulating serotonin concentrations, highlighting the microbiota’s role in serotonin homeostasis [134]. Additionally, some bacteria are capable of de novo neurotransmitter production. For example, strains of *Bifidobacterium* and *Lactobacillaceae* can produce GABA, the main inhibitory neurotransmitter in the CNS [135].

Bacterial metabolites play a crucial role in GBA communication. In the distal intestine, SCFAs are produced through microbial fermentation of non-digestible polysaccharides, such as resistant starches and dietary fibres [136]. The primary SCFAs, butyrate, propionates and acetate, constitute approximately 95% of the total SCFAs in the gut [136]. Specific substrates such as acetogenic fibres, including inulin and galactooligosaccharides, promote the production of acetate via enteric bacteria such as *Prevetolla* spp., *Akkermansia muciniphila*, or *Bacteroidota* phyla spp. [137,138,139]. Both *Bacteroidota* and *Bacillota* phyla contribute to propionate production, while butyrate is predominantly produced by species within the *Bacillota* phylum. To a lesser extent, members of the *Pseudomonadota*, *Actinomycetota*, and *Fusobacteriota* phyla also participate in butyrate production [140,141].

In addition to their metabolic functions, SCFAs are involved in the regulation of inflammatory pathways and neuroinflammation [142,143,144]. SCFAs can influence hormonal secretions from enteroendocrine cells, including the YY peptide, the glucagonoid peptide, and cholecystokinin, thereby impacting gut–brain communication [145]. Furthermore, SCFAs can cross the BBB and move to the CNS, inducing the modification of neurotrophic factors and altering the levels of many neurotransmitters, lastly affecting neurotransmission and brain function [146,147,148].

**Figure 2:**
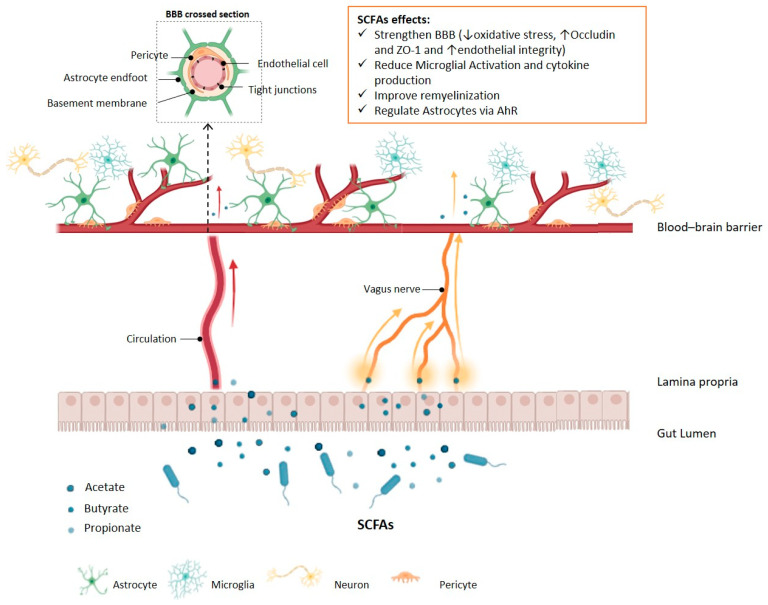
Short-chain fatty acid effects on blood–brain barrier integrity, microglia, and astrocyte regulation. SCFAs, including acetate, butyrate, and propionate, are produced in the gut lumen by microbial fermentation and reach the lamina propria. From there, SCFAs enter circulation or signal via the vagus nerve to exert central nervous system effects. SCFAs strengthen the BBB by reducing (↓) oxidative stress, increasing (↑) expression of tight junction proteins (Occludin, ZO-1), and enhancing (↑) endothelial integrity. Additionally, SCFAs reduce microglial activation and cytokine production, promote remyelination, and regulate astrocyte function through aryl hydrocarbon receptor (AhR) signalling, collectively contributing to neuroprotection and homeostasis. The BBB structure includes endothelial cells, tight junctions, pericytes, astrocyte endfeet, and the basement membrane, all of which are targets for SCFA-mediated modulation. SCFAs: short-chain fatty acids, BBB: blood–brain barrier, ZO-1 = zonula occludens-1.

The significant role of SCFAs in maintaining the BBB integrity is evidenced in studies using GF animal models, which exhibit high BBB permeability. This effect appears to be reversible given that the repopulation of sterile experimental animals with SCFA-generating bacterial species can improve endothelial integrity [149,150]. Furthermore, propionate has been shown to protect the BBB from oxidative stress by changing its permeability after exposure to lipopolysaccharides (LPSs) [151]. Butyrate acts in re-establishing BBB permeability and inducing a neuroprotective effect by enhancing the expression of tight junction proteins, such as occludin and zonula occludens, as observed in a brain injury model [152]. Once in circulation, serum butyrate can reach the CNS and exert positive neurological effects [153] (Figure 2).

Some studies have shown that orally administered butyrate can reduce anxiety and depression caused by chemotherapeutic agents and induce anti-inflammatory and neuroprotective effects in mice [153]. Furthermore, butyrate has been reported to improve remyelination and attenuate neuroinflammation associated with a high-fat diet in the cerebral cortex [154]. High-fat diets are known to trigger oxidative stress and hypothalamic inflammation, contributing to neurodegenerative processes [155,156]. In general, decreased levels of intestinal SCFAs, such as butyrate, are typically observed in disease models correlated with intestinal dysbiosis, which can promote the expansion of neuroinflammation and, subsequently, neurological disorders [155,156]. Although butyrate supplementation has shown potential in mitigating dysbiosis-related complications, restoring physiological butyrate concentrations in the gut remains a challenge. It is worth noting that the mechanism by which butyrate affects neural physiology and behaviour is still poorly understood [157].

Gut barrier damage induces the access of microbial components such as LPSs to the systemic circulation [158,159]. LPSs may move to the brain and activate astrocytes and microglia, thus enhancing cytokine generation and neuroinflammation. Elevated circulating LPS levels stimulate microglia in many regions of the brain, leading to synaptic loss and neuronal damage [158,159]. Chronic pro-inflammatory signals released by activated microglia may turn astrocytes into neurotoxic reactive cells, leading to the degeneration of neurons and oligodendrocytes [158,159]. Additionally, the missing or reduced diversity of the GM impairs microglia maturation and their ability to elicit effective immune responses [11].

SCFAs can mitigate microglial cell activation and pro-inflammatory cytokine production. For example, in microglial cells, butyrate promotes anti-inflammatory phenotypes by inducing morphological modifications, including the elongation of cellular protrusions and the restoration of their normal characteristic form [160,161]. It is worth noting that the oral administration of butyrate, acetate, and propionate can support microglial repair processes [11].

In addition, bacteria metabolites can also exert a regulatory effect in astrocytes via the aryl hydrocarbon receptor (AhR). In detail, the GM metabolizes tryptophan into AhR agonists, such as indoles, stimulating AhR signalling and decreasing neuroinflammation in experimental models of autoimmune encephalomyelitis [162]. Additionally, studies reported a relationship between the GM and myelination by modulating myelination gene expression and myelin protein levels in the prefrontal cortex of sterile animals [163].

## 5. Gut Microbiota and NSs

### 5.1. GM Role in Neurosteroidogenesis

Given that genetic material is heritable, it is worthwhile to explore the genetic material that is passed down in our lineage through a multitude of genetic pathways over evolutionary timescales and the acquisition of properties that promote host health and fitness [164]. For example, a bacterial metabolite called ‘coprostanol’ synthesized in the GI tract from cholesterol plays a crucial role in the regulation of serum cholesterol levels. In fact, cholesterol released into the gut as a constituent of bile is generally reabsorbed in the small intestine, accessing the circulation with nutrients that improve its emulsification. However, when some enteric microbes convert cholesterol to coprostanol, they avoid its reabsorption and therefore, decrease the serum cholesterol level [164]. In a study by Kenny et al., coprostanol was present in almost all urine samples of wildtype vertebrate species and in four out of five humans [164]. Given that humans who lack coprostanol-producing microbes are susceptible to elevated serum cholesterol levels, these microbes are likely to be important in maintaining normal cholesterol levels and preventing atherosclerosis [165]. Coprostanol production varies over time and may be modulated by interventions that modify the composition of the GM [165]. This model that relates the extinction of a primitive and essential bacterial gene in the intestine to hypercholesterolemia is still valid and relevant to neurological disorders [166]. Generally, the body tends to select GM taxa for the fabrication of inhibitory neuromodulators, such as NSs or their precursors. Subsequently, the lack of such species and their metabolites can lead to psychiatric pathology [166]. Indeed, many NS alterations were noticed in male GF mice, confirming the close relationship between the GM and steroidogenesis in the CNS [31]. Furthermore, it was reported that GM modifications can also affect NSs in the brain, strengthening the hypothesis of the microbiota-mediated regulation of neuroendocrine pathways in the brain [90].

Under specific conditions, the GM may generate interesting secondary metabolites, such as NSs and mainly para-endogenous anxiolytics, that can be familiar or novel [166]. Para-endogenous NSs can be described as compounds that exist in the circulation at subtherapeutic levels and can be concentrated in synaptic vesicles to achieve efficient concentrations within the CNS [166]. The range of para-endogenous NS concentrations can fluctuate inside a given population according to many aspects, including antibiotic use and food regimens [166]. Cholesterol is the main precursor of corticoids, bile salts, and steroid sex hormones. All of these compounds go through enterohepatic circulation in a similar way and can communicate with the GM [166].

### 5.2. Role of NSs in Modulating GM

Recent investigations supported the notion that host-produced steroids interconnect with human-associated bacteria; however, the mechanisms underlying these interactions and their physiological effects remain vague [16]. Mccury et al. documented that the human gut bacteria *Eggerthella lenta* and *Gordonibacter pamelaeae*, which exist in high prevalence and low abundance, convert abundant biliary corticoids into progestins by 21-dehydroxylation, thus converting a class of immunoregulatory and metabo-regulatory steroids into a class of NSs and sex hormones deemed as high-potency secondary metabolites [16]. Indeed, they convert tetrahydrodeoxycorticosterones to tetrahydroprogesterones, that is, pregnanolones. They also identified a group of bacterial genes, specified as Elen_2451–2454, that conducts 21-dehydroxylation, which is an enzymatic activity carried out exclusively by microbes [16]. In addition, they fortuitously revealed the great impact of the GM on promoting reductive 21-dehydroxylation and the transformation of abundant glucocorticoids present in human bile, especially THDOC, into progestins, namely tetrahydroprogesterones (THPs), through the production of hydrogen gas that regulates the secondary metabolism in the gut [16]. The increased levels of some bacterial progestins, such as allopregnanolone, commonly known as brexanolone, a positive allosteric modulator of GABA_A_ and FDA-approved treatment for postpartum depression, are found in pregnant women’s feces [16]. This drug is used to maintain the fetus in a quasi-sedated state throughout the third trimester of pregnancy [167]. ALLO can also induce negative allosteric modulatory effects on the 5HT3 receptor: its properties and nicotinic acetylcholine receptors can be determinants for maternal psychiatric health, given that decreased levels of allopregnanolone in late pregnancy are associated with the likelihood of postpartum depression [167]. Thus, the bacterial transformation of corticoids into progestins can impact the host’s physiology, especially with respect to pregnancy and women’s health [16]. In addition, McCurry et al. reported that ISOALLO was used in clinical trials for Tourette syndrome therapy and mentioned that the GM contributes to the production of these neuroactive progestin metabolites [16]. These metabolites can modify the activity of membrane-bound GABA and NMDA receptors in gut-accessing sensory neurons and thus, modulate the host’s neurological signalling pathways [16].

## 6. Therapeutic Perspectives

### 6.1. GM Based Approaches to Treat Neuroinflammation

Brain-derived GABA, as well as peripheral GABA systems, exert a powerful modulation on brain function through their impact on the GI tract and specifically on the GM. Given that GABA cannot cross the BBB, it is likely that peripherally generated GABA affects brain function indirectly [168,169,170]. NSs also exert non-genomic effects by interacting with receptors such as GABA_A_, which mediates the primary inhibitory neurotransmission in the brain [135]. This evidence supports the potential of GABA-based dietary interventions to regulate brain function. Dietary interventions aimed at maintaining GABA homeostasis are essential to improve therapeutic outcomes across the BGM axis [171]. Innovative approaches, such as probiotics and prebiotics, could represent promising strategies for the treatment of neurodegenerative diseases and psychiatric disorders.

NSs are present not only in the CNS but also in the GI tract, where elevated levels of these potent neuroactive modulators have been suggested as peripheral influences on brain function [172]. Beyond the positive allosteric modulators (PAMs) of GABA_A_ receptors, other bacterial metabolites in the periphery may also modulate brain activity. The integration of the biophysical and structural models of individual GABA_A_ receptors coupled with p bacterial metabolomic data has enabled the identification of new endogenous compounds that could potentially modulate GABA_A_ receptor function, providing new avenues for drug discovery [172].

A growing body of evidence has identified bacterial metabolites, in addition to GABA, that influence GABA_A_ receptors and transmit beneficial effects [172]. A plausible approach to naturally benefit from such signalling pathways may be to provoke their generation indirectly with agents that modulate the GM. Dietary interventions based on the use of probiotics, prebiotics, or a symbiotic combination of both, could be an effective approach [173]. The acceptance of these formulations is continually growing due to their obvious beneficial effect on GI homeostasis in humans [171]. Significant preclinical and clinical research has focused on GABA-based probiotic interventions. These preparations are commonly available in the form of supplements containing GABA-producing bacteria or fermented foods/beverages [174].

Among these, *Lactobacillus* and *Bifidobacterium* are reported to be the most frequently used genera of GABA-producing microorganisms in commercially available dietary interventions [172]. Such GABA-enriched dietary products have shown measurable improvements in psychiatric conditions such as depression [175,176,177], anxiety [56,119,178,179], cognition [180], and schizophrenia [181], as well as neurological disorders such as pain [182] and epilepsy [183].

GABA seems to play a crucial role in the health-promoting activity of some prebiotics that can be consumed alone or together with probiotic strains such as *L. plantarum* [184]. The most common prebiotics are within the polysaccharide group and contain inulin, fructooligosaccharides, galactooligosaccharides, and human milk oligosaccharides [185,186].

The positive effects of prebiotics on the gut barrier are mediated by promoting the abundance of definite microbiota strains and, as such, the levels of their metabolic products [187,188]. These preparations, which include GABA and SCFAs, upgrade classical probiotics composed primarily of the genera *Lactobacillus* and *Bifidobacterium* [187,188]. In addition to the *genera Lactobacillus* and *Bifidobacterium* qualified as the most eminent GABA producers in the food industry, the genera *Bacteroides* and *Eubacterium* can also be utilized as abundance-promoters of GABA-producing probiotic strains. These genera include inulin-degrading species that may indirectly contribute to the preservation of a healthy gut [189]. Furthermore, an in vitro study using a gut model demonstrated that treatment with certain human milk oligosaccharides, alone or in different combination mixtures, increased GABA levels in the stool of both children and adults [190]. Duranti et al. demonstrated that human milk oligosaccharides improved GABA production, which is probably mediated by an increase in special microbial species such as *Bifidobacterium adolescentis* [188]. They also noted a significant correlation between GABA and *Bacteroides* species, using fructose molecules called fructans as prebiotics, suggesting that the prebiotic exerts a selective action on the intestinal microbiota profile [191]. This study highlights specific prebiotic combinations that could maximize health benefits by promoting GABA production through targeted microbial species. Furthermore, a recent in vitro human investigation reported a significant physiological increase in GABA synthesis after combined oligofructose and 2′-fucosyllactose supplementations [192].

Interestingly, polyphenols represent another relevant class of plant-derived prebiotics linked to various health benefits, including mental health [193,194]. These compounds may serve as targeted GABA-based dietary interventions, either directly by promoting GABA-producing species or indirectly by improving their enzymatic abilities, such as the bacterial vitamin B6, which is essential for GABA synthesis [191,194,195,196]. Recent studies have noticed that bacteria within the human gut [135] can indirectly boost the emergence of other bacteria, showing mutualistic interactions between gut bacteria that are classified as crucial for a healthy gut ecosystem [192,197,198]. Thus, the effect of GABA on the BGM axis could be likely both directly and indirectly modulated.

### 6.2. NSs to Treat Neuroinflammatory Conditions

Many CNS diseases driven by neuroinflammation are associated with an alteration in NS levels, emphasizing their substantial role in preserving tissue homeostasis. Therefore, restoring NS levels to their original state can be deemed as a potential therapeutic strategy. Several studies have investigated both endogenous NSs and their analogues [26].

ALLO administration has been shown to exert beneficial effects in in vitro or in vivo models. In rodents, the stabilization of ALLO levels in microglial cells induced anti-inflammatory properties, improved microglial migratory ability, and decreased the phagocytosis of oligodendrocytes [199]. Additionally, ALLO supported microglial survival as a protective response against the oxidative damage caused by rotenone [200]. In experimental autoimmune encephalomyelitis (EAE) mice, ALLO treatments significantly reduced inflammatory response, microglia reactivity, demyelination, axonal injury, and lymphocyte infiltration [7].

Many in vitro and in vivo investigations underscored the protective role of progesterone uptake. In fact, in hypoxic primary microglia, progesterone administration decreased the inflammatory response and favoured anti-inflammatory gene expression [201]. Similarly, other investigations conducted in male rodents noticed that early progesterone treatments following a traumatic brain injury induced a decrease in inflammatory response and microgliosis [202,203]. In the female EAE mouse model, progesterone treatments reduced inflammation, demyelination, and cell infiltration, which subsequently delayed disease onset and progression of the disease [204]. Similarly, in LPS-stimulated microglia, progesterone treatments decreased the inflammatory response [99].

In male and female animals, oestrogens, especially estriol treatments, manifested their prominent neuroprotective and anti-inflammatory effects, including decreased inflammatory cytokines, microgliosis, and demyelination alongside increased expressions of anti-inflammatory markers in microglia, as well as the enhanced survival of neurons and oligodendrocytes [205].

Finally, DHEA has been reported to effectively decrease microglia-mediated neuroinflammation both in vitro and in vivo, particularly in models of brain inflammation caused by LPS-stimulated microglia [104]. Similar anti-inflammatory effects in the CNS were also observed in female EAE mice across different studies [206,207,208].

In general, the neuromodulatory effects that enhance GABAergic transmission support their beneficial role in treating neurological disorders [209]. In fact, they have been shown to be effective against glutamate toxicity, ischemia, epilepsy, and acute and traumatic brain injury [209]. Some examples of key NSs, their modes of action, and their therapeutic roles in different disease models are summarized in Table 3 [209].

Despite their efficiency in reducing neuroinflammation, NSs have limitations such as short half-lives, low bioavailability, and adverse pharmacokinetics [4,26]. To overcome these issues, synthetic NSs analogues have been developed with the advantage of decreased metabolism and a preserved activity as therapeutic agents regulating GABA_A_ receptors. A synthetic analogue of ALLO called ‘Ganaxolone’ shows a stable structure that prevents endogenous NSs disadvantages and provides reduced microglial activation and remyelination in ovariectomized EAE rats [219,220].

NSs exert their neuromodulatory effects by binding to specific allosteric sites on GABA_A_ receptors, thus modulating inhibitory signalling pathways that influence behaviour and mood regulation. While endogenous NSs finely tune these processes under physiological conditions, exogenous NSs and their analogues are increasingly employed in clinical settings, including as general anesthetics and anti-convulsants [221]. However, despite their promising therapeutic profiles, both natural and synthetic NSs can induce side effects primarily linked to their CNS depressant activity. These adverse effects may include dizziness, sedation, fatigue, cognitive impairment, and, less commonly, respiratory depression and hypotension [221,222]. In detail, Ganaxolone has been associated with dose-dependent somnolence and dizziness in clinical trials, though these effects are generally mild to moderate and manageable with dose titration [223]. Notably, these side effects are typically less severe than those observed with traditional anesthetics or benzodiazepines, contributing to a more favourable safety and tolerability profile [221,224]. Nevertheless, careful monitoring is warranted, especially in populations vulnerable to CNS depression, such as the elderly or patients with respiratory compromise. Further clinical studies are ongoing to better define the long-term safety of these compounds and optimize dosing strategies to maximize benefit while minimizing adverse effects.

## 7. Conclusions

In summary, both preclinical and clinical studies highlight the potential of neurosteroids to reduce neuroinflammation and improve outcomes in neurological and psychiatric disorders. These findings suggest that NSs may offer therapeutic options for patients who do not respond to standard treatments [225] and, at the same time, that the gut microbiota has emerged as a key regulator of host metabolism, immune signalling, and nervous system function [226]. By influencing biochemical pathways, gut microbes can shape the production and activity of the endogenous signalling molecules, NSs. NSs exert rapid, non-genomic effects, partly through receptors like GABA_A_ [225], the primary inhibitory neurotransmitter system [135]. The interaction with the gut–brain axis represents a promising field with significant therapeutic potential. However, more targeted and well-designed clinical trials are needed to clarify the mechanisms underlying the NS–GM relationship and translate these insights into effective treatments.

## 8. Search Strategy

We conducted a comprehensive literature search to identify relevant studies exploring the interactions between neurosteroids, gut microbiota, and neuroinflammation. The focus was on in vivo, in vitro, ex vivo, and cellular models, as well as clinical studies investigating mechanistic insights and potential therapeutic strategies within this field. The search was performed using the PubMed database, with the aim of ensuring a precise, high-quality, and up-to-date collection of scientific publications. To optimize the search process, we utilized the Medical Subject Headings (MeSH) system combined with relevant free-text keywords to capture both indexed and nonindexed articles. The primary MeSH terms selected were as follows: “Gut Microbiota” [MeSH], “Neurosteroids” [MeSH], and “Neuroinflammation” [MeSH]. To enhance the specificity of the search and ensure a comprehensive coverage of studies addressing the intersection of these domains, we applied the following Boolean operator combinations: (“Gut Microbiota” [MeSH]) AND (“Neurosteroids” [MeSH]); (“Neurosteroids” [MeSH]) AND (“Neuroinflammation” [MeSH]); as well as (“Gut Microbiota” [MeSH]) AND (“Neuroinflammation” [MeSH]). In addition to MeSH terms, free text keywords were used to broaden the scope of the search and include recent or emerging literature not yet indexed under specific headings. These keywords included the microbiota, gut microbiome, gut–brain axis, neurosteroids, neuroactive steroids, neuroinflammation, neurodegeneration, microbiota-derived metabolites, neuroimmune interactions. The searches were limited to publications in English and no restrictions were applied on the publication date to ensure historical and foundational studies were also considered. However, particular emphasis was placed on articles published within the last five years to capture recent advancements in this rapidly evolving field. Relevant references from identified articles were also manually screened to ensure the inclusion of additional key studies not captured by the database search (that is, the snowball approach). The final selection included original research articles, reviews, and meta-analyses that provided mechanistic insights or discussed therapeutic perspectives related to neurosteroids, gut microbiota, and neuroinflammation in both physiological and pathological contexts, with particular relevance to neurodegenerative diseases such as Alzheimer’s disease, multiple sclerosis, and amyotrophic lateral sclerosis.

## Figures and Tables

**Table 1:** Classification of neurosteroids (NSs) according to their chemical structure.

NS Type	Examples	Effects	References
Pregnane NS	- ALLO - PREG - ISOALLO - Epipregnanolone - THDOC	- decreases stress - improves GABAergic neurotransmission - anxiolytic	[16,26,27]
Androstane NS	- Androstanediol - Etiocholanone	- reduces stress - promotes resilience in humans - attenuates emotional reactivity - anxiolytic - antidepressant	[28,29,30]
Sulfated NS	- PS - DHEAS	- antidepressant	[30]

**Table 2:** Classification of NSs according to their action on receptors.

NS Type	Action on Receptors	Effects	References
Inhibitory NS	GABA_A_ receptor positive modulator	- anxiolytic - stress reducing - antidepressant - prosocial - rewarding - prosexual - antiaggressive - prosleep - sedative - cognitive and memory improvement - anesthetic - analgesic - anticonvulsant - neurogenic - neuroprotective	[43,45]
Excitatory NS	- GABA_A_ receptor negative modulators - NMDA receptor weak positive modulators - sigma-1 receptor agonists	- cognitive and memoryenhancing - anxiogenic - antidepressant - convulsant - neurogenic - neuroprotective	[46]

**Table 3:** Relevant clinical studies reporting therapeutic roles of neurosteroids in different diseases. NSs = Neurosteroids; RCT = Randomized Controlled Trial.

Key NSs	Mode of Action	Therapeutic Roles	Study Type and Participants	Indications/ Diseases	Side Effects
**Brexanolone**	Positive allosteric modulator of synaptic and extrasynaptic GABA_A_ receptors	- Rapid improvement in depressive symptoms - Reduction in anxiety and insomnia symptoms	- Randomized Controlled Trial (RCT), *n* = 21 brexanolone, 21 placebos [210] - Pooled analysis of 3 Double-Blind, Placebo-Controlled RCTs, *n* ≈ 209 [211]	Postpartum depression	Dizziness, somnolence
**Zuranolone**	Positive allosteric modulator of synaptic and extrasynaptic GABA_A_ receptors	- Rapid reduction in depressive symptoms	- Post hoc Analysis from Double-Blind RCTs, *n* not specified [212] - Double-Blind, Placebo-Controlled RCT, *n* = 89 [213] - Phase 1, Double-Blind, Placebo-Controlled Study, *n* = 60 [214]	Postpartum depression, major depressive disorder	Favourable safety profile; no serious adverse events or deaths
**Sepranolone**	Modulator of GABA_A_ receptors	- Alleviation of negative mood symptoms - Reduction in emotional distress	- Randomized, Double-Blind, Placebo-Controlled Study, *n* = 206 [215]	Premenstrual dysphoric disorder	No serious adverse effects
**Ganaxolone**	Positive allosteric modulator of GABA_A_ receptors	- Adjunctive therapy for depression in postmenopausal women - Effective monotherapy for refractory status epilepticus - Reduction in seizure frequency in PCDH19-clustering epilepsy	- Open-Label Pilot Study, *n* = 12 [216] - Open-Label, Dose-Finding, Phase 2 Trial, *n* = 17 [217] - Phase 2, Placebo-Controlled Study, *n* = 101 [218]	Depression in postmenopausal women, refractory status epilepticus, PCDH19-clustering epilepsy	Somnolence

## Abbreviations

The following abbreviations are used in this manuscript:

GBA	Gut–brain axis
GF	Germ free
GM	GutMicrobiota
CNS	Central nervous system
NS	Neurosteroid
EAE	Experimental Autoimmune Encephalomyelitis
BBB	Blood–brain barrier
DHEA	Dehydroepiandrosterone
SCFAs	Short-chain fatty acids
ISOALLO	Isoallopregnanolone
ALLO	Allopregnanolone
THDOC	Allotetrahydrodeoxycorticosterone
GABA_A_	Gamma-aminobutyric acid type A
NMDA	N-methyl-D-aspartate
DHEAS	Dehydroepiandrosterone sulphate
StAR	Steroidogenic acute regulatory protein
PREG	pregnanolone
ALS	Amyotrophic lateral sclerosis
PS	Pregnenolone sulfate

## References

[1] Carabotti M., Scirocco A., Maselli M.A., Severi C. (2015). The gut-brain axis: Interactions between enteric microbiota, central and enteric nervous systems. Ann. Gastroenterol..

[2] Cryan J.F., O’Riordan K.J., Cowan C.S.M., Sandhu K.V., Bastiaanssen T.F.S., Boehme M., Codagnone M.G., Cussotto S., Fulling C., Golubeva A.V. (2019). The Microbiota-Gut-Brain Axis. Physiol. Rev..

[3] Baulieu E.E., Robel P. (1990). Neurosteroids: A new brain function?. J. Steroid Biochem. Mol. Biol..

[4] Reddy D.S., Estes W.A. (2016). Clinical Potential of neurosteroids for CNS Disorders. Trends Pharmacol. Sci..

[5] Balan I., Beattie M.C., O’Buckley T.K., Aurelian L., Morrow A.L. (2019). Endogenous Neurosteroid (3α,5α)3-Hydroxypregnan-20-one Inhibits Toll-like-4 Receptor Activation and Pro-inflammatory Signaling in Macrophages and Brain. Sci. Rep..

[6] Giatti S., Boraso M., Melcangi R.C., Viviani B. (2012). Neuroactive steroids, their metabolites, and neuroinflammation. J. Mol. Endocrinol..

[7] Noorbakhsh F., Ellestad K.K., Maingat F., Warren K.G., Han M.H., Steinman L., Baker G.B., Power C. (2011). Impaired neurosteroid synthesis in multiple sclerosis. Brain.

[8] Smith C.D., Wekstein D.R., Markesbery W.R., Frye C.A. (2006). 3α,5α-THP: A Potential Plasma Neurosteroid Biomarker in Alzheimer’s Disease and Perhaps Non-Alzheimer’s Dementia. Psychopharmacology.

[9] Di Michele F., Longone P., Romeo E., Lucchetti S., Brusa L., Pierantozzi M., Bassi A., Bernardi G., Stanzione P. (2003). Decreased Plasma and Cerebrospinal Fluid Content of Neuroactive Steroids in Parkinson’s Disease. Neurol. Sci..

[10] Lucchi C., Simonini C., Rustichelli C., Avallone R., Zucchi E., Martinelli I., Biagini G., Mandrioli J. (2024). Reduced Levels of neurosteroids in Cerebrospinal Fluid of Amyotrophic Lateral Sclerosis Patients. Biomolecules.

[11] Erny D., Hrabe de Angelis A.L., Jaitin D., Wieghofer P., Staszewski O., David E., Keren-Shaul H., Mahlakoiv T., Jakobshagen K., Buch T. (2015). Host microbiota constantly control maturation and function of microglia in the CNS. Nat. Neurosci..

[12] Sharon G., Sampson T.R., Geschwind D.H., Mazmanian S.K. (2016). The Central Nervous System and the Gut Microbiome. Cell.

[13] Dalile B., Van Oudenhove L., Vervliet B., Verbeke K. (2019). The role of short-chain fatty acids in microbiota–gut–brain communication. Nat. Rev. Gastroenterol. Hepatol..

[14] Agus A., Planchais J., Sokol H. (2018). Gut Microbiota Regulation of Tryptophan Metabolism in Health and Disease. Cell Host Microbe.

[15] Vagnerová K., Gazárková T., Vodička M., Ergang P., Klusoňová P., Hudcovic T., Šrůtková D., Petr Hermanová P., Nováková L., Pácha J. (2024). Microbiota modulates the steroid response to acute immune stress in male mice. Front. Immunol..

[16] McCurry M.D., D’Agostino G.D., Walsh J.T., Bisanz J.E., Zalosnik I., Dong X., Morris D.J., Korzenik J.R., Edlow A.G., Balskus E.P. (2024). Gut bacteria convert glucocorticoids into progestins in the presence of hydrogen gas. Cell.

[17] Org E., Mehrabian M., Parks B.W., Shipkova P., Liu X., Drake T.A., Lusis A.J. (2016). Sex differences and hormonal effects on gut microbiota composition in mice. Gut Microbes.

[18] Santos-Marcos J.A., Barroso A., Rangel-Zuniga O.A., Perdices-Lopez C., Haro C., Sanchez-Garrido M.A., Molina-Abril H., Ohlsson C., Perez-Martinez P., Poutanen M. (2020). Interplay between gonadal hormones and postnatal overfeeding in defining sex-dependent differences in gut microbiota architecture. Aging.

[19] Jaggar M., Rea K., Spichak S., Dinan T.G., Cryan J.F. (2020). You’ve got male: Sex and the microbiota-gut-brain axis across the lifespan. Front. Neuroendocr..

[20] Sze Y., Brunton P.J. (2022). Neurosteroids and early-life programming: An updated perspective. Curr. Opin. Endocr. Metab. Res..

[21] Biagini G., Panuccio G., Avoli M. (2010). Neurosteroids and epilepsy. Curr. Opin. Neurol..

[22] Reddy D.S. (2010). Neurosteroids: Endogenous role in the human brain and therapeutic potentials. Prog. Brain Res..

[23] Yoon S.Y., Roh D.H., Seo H.S., Kang S.Y., Moon J.Y., Song S., Beitz A.J., Lee J.H. (2010). An increase in spinal dehydroepiandrosterone sulfate (DHEAS) enhances NMDA-induced pain via phosphorylation of the NR1 subunit in mice: Involvement of the sigma-1 receptor. Neuropharmacology.

[24] Belelli D., Lambert J.J. (2005). Neurosteroids: Endogenous regulators of the GABA(A) receptor. Nat. Rev. Neurosci..

[25] Penke B., Fulop L., Szucs M., Frecska E. (2018). The Role of Sigma-1 Receptor, an Intracellular Chaperone in Neurodegenerative Diseases. Curr. Neuropharmacol..

[26] Yilmaz C., Karali K., Fodelianaki G., Gravanis A., Chavakis T., Charalampopoulos I., Alexaki V.I. (2019). Neurosteroids as regulators of neuroinflammation. Front. Neuroendocr..

[27] Henderson V.W. (2018). Progesterone and human cognition. Climacteric.

[28] Almeida F.B., Pinna G., Barros H.M. (2021). The Role of HPA Axis and Allopregnanolone on the Neurobiology of Major Depressive Disorders and PTSD. Int. J. Mol. Sci..

[29] Ben Dor R., Marx C.E., Shampine L.J., Rubinow D.R., Schmidt P.J. (2015). DHEA metabolism to the neurosteroid androsterone: A possible mechanism of DHEA’s antidepressant action. Psychopharmacology.

[30] Sripada R.K., Marx C.E., King A.P., Rajaram N., Garfinkel S.N., Abelson J.L., Liberzon I. (2013). DHEA enhances emotion regulation neurocircuits and modulates memory for emotional stimuli. Neuropsychopharmacology.

[31] Diviccaro S., Caputi V., Cioffi L., Giatti S., Lyte J.M., Caruso D., O’Mahony S.M., Melcangi R.C. (2021). Exploring the Impact of the Microbiome on Neuroactive Steroid Levels in Germ-Free Animals. Int. J. Mol. Sci..

[32] Liang J.J., Rasmusson A.M. (2018). Overview of the molecular steps in steroidogenesis of the GABAergic neurosteroids allopregnanolone and pregnanolone. Chronic Stress.

[33] Miller W.L. (2017). Disorders in the initial steps of steroid hormone synthesis. J. Steroid Biochem. Mol. Biol..

[34] Fernández C., del Val T Lobo M., Gómez-Coronado D., Lasunción M.A. (2004). Cholesterol is essential for mitosis progression and its deficiency induces polyploid cell formation. Exp. Cell. Res..

[35] Rone M.B., Fan J., Papadopoulos V. (2009). Cholesterol transport in steroid biosynthesis: Role of protein-protein interactions and implications in disease states. Biochim. Biophys. Acta.

[36] Castillo A.F., Orlando U., Helfenberger K.E., Poderoso C., Podesta E.J. (2015). The role of mitochondrial fusion and StAR phosphorylation in the regulation of StAR activity and steroidogenesis. Mol. Cell. Endocrinol..

[37] Papadopoulos V., Aghazadeh Y., Fan J., Campioli E., Zirkin B., Midzak A. (2015). Translocator protein-mediated pharmacology of cholesterol transport and steroidogenesis. Mol. Cell. Endocrinol..

[38] Mellon S.H., Griffin L.D. (2002). Neurosteroids: Biochemistry and clinical significance. Trends Endocrinol. Metab..

[39] Porcu P., Barron A.M., Frye C.A., Walf A.A., Yang S.Y., He X.Y., Morrow A.L., Panzica G.C., Melcangi R.C. (2016). Neurosteroidogenesis today: Novel targets for neuroactive steroid synthesis and action and their relevance for translational research. J. Neuroendocr..

[40] Thomas J.L., Bose H.S. (2015). Regulation of human 3-beta-hydroxysteroid dehydrogenase type-2 (3βHSD2) by molecular chaperones and the mitochondrial environment affects steroidogenesis. J. Steroid Biochem. Mol. Biol..

[41] Mondragón J.A., Serrano Y., Torres A., Orozco M., Segovia J., Manjarrez G., Romano M.C. (2021). Glioblastoma cells express crucial enzymes involved in androgen synthesis: 3β-hydroxysteroid dehydrogenase, 17-20α-hydroxylase, 17β-hydroxysteroid dehydrogenase and 5α-reductase. Endocrinol. Diabetes Metab..

[42] Baulieu E.E., Fuxe K., Gustafsson J.Å., Wetterberg L. (1981). Steroid hormones in the brain: Several mechanisms?. Steroid Hormone Regulation of the Brain.

[43] Diviccaro S., Cioffi L., Falvo E., Giatti S., Melcangi R.C. (2022). Allopregnanolone: An overview on its synthesis and effects. J. Neuroendocr..

[44] Maitra R., Reynolds J.N. (1999). Subunit dependent modulation of GABA_A_ receptor function by neuroactive steroids. Brain Res..

[45] Morris K.D., Moorefield C.N., Amin J. (1999). Differential modulation of the gamma-aminobutyric acid type C receptor by neuroactive steroids. Mol. Pharmacol..

[46] Morales-Lázaro S.L., González-Ramírez R., Rosenbaum T. (2019). Molecular interplay between the sigma-1 receptor, steroids, and ion channels. Front. Pharmacol..

[47] Paoletti P., Neyton J. (2007). NMDA receptor subunits: Function and pharmacology. Curr. Opin. Pharmacol..

[48] Vaudry H., Ubuka T., Soma K.K., Tsutsui K. (2022). Editorial: Recent progress and perspectives in neurosteroid research. Front. Endocrinol..

[49] Stell B.M., Brickley S.G., Tang C.Y., Farrant M., Mody I. (2003). Neuroactive steroids reduce neuronal excitability by selectively enhancing tonic inhibition mediated by delta subunit-containing GABA_A_ receptors. Proc. Natl. Acad. Sci. USA.

[50] Akk G., Covey D.F., Evers A.S., Steinbach J.H., Zorumski C.F., Mennerick S. (2007). Mechanisms of neurosteroid interactions with GABA(A) receptors. Pharmacol. Ther..

[51] Akk G., Bracamontes J.R., Covey D.F., Evers A., Dao T., Steinbach J.H. (2004). Neuroactive steroids have multiple actions to potentiate GABA_A_ receptors. J. Physiol..

[52] Shu H.J., Eisenman L.N., Jinadasa D., Covey D.F., Zorumski C.F., Mennerick S. (2004). Slow actions of neuroactive steroids at GABA_A_ receptors. J. Neurosci..

[53] Ambert N., Greget R., Haeberlé O., Bischoff S., Berger T.W., Bouteiller J.M., Baudry M. (2010). Computational studies of NMDA receptors: Differential effects of neuronal activity on efficacy of competitive and non-competitive antagonists. Open Access Bioinform..

[54] Temme L., Schepmann D., Schreiber J.A., Frehland B., Wünsch B. (2018). Comparative pharmacological study of common NMDA receptor open channel blockers regarding their affinity and functional activity toward GluN2A and GluN2B NMDA receptors. ChemMedChem.

[55] Ratner M.H., Kumaresan V., Farb D.H. (2019). Neurosteroid actions in memory and neurologic/neuropsychiatric disorders. Front. Endocrinol..

[56] Bravo J.A., Forsythe P., Chew M.V., Escaravage E., Savignac H.M., Dinan T.G., Bienenstock J., Cryan J.F. (2011). Ingestion of lactobacillus strain regulates emotional behavior and central GABA receptor expression in a mouse via the vagus nerve. Proc. Natl. Acad. Sci. USA.

[57] Monteagudo-Mera A., Fanti V., Rodriguez-Sobstel C., Gibson G., Wijeyesekera A., Karatzas K.A., Chakrabarti B. (2023). Gamma aminobutyric acid production by commercially available probiotic strains. J. Appl. Microbiol..

[58] Bitran D., Hilvers R.J., Kellogg C.K. (1991). Anxiolytic effects of 3α-hydroxy-5α[β]-pregnan-20-one: Endogenous metabolites of progesterone that are active at the GABA_A_ receptor. Brain Res..

[59] Khisti R.T., Chopde C.T., Jain S.P. (2000). Antidepressant-like effect of the neurosteroid 3α-hydroxy-5α-pregnan-20-one in mice forced swim test. Pharmacol. Biochem. Behav..

[60] Johasson I.M., Birzniece V., Lindblad C., Olsson T., Backstrom T. (2002). Allopregnanolone inhibits learning in the Morris water maze. Brain Res..

[61] Frye C.A., Bayon L.E., Pursnani N.K., Purdy R.H. (1998). The neurosteroids, progesterone and 3α,5α-THP, enhance sexual motivation, receptivity, and proceptivity in female rats. Brain Res..

[62] Finn D.A., Phillips T.J., Okorn D.M., Chester J.A., Cunningham C.L. (1997). Rewarding effect of the neuroactive steroid 3α-hydroxy-5α-pregnan-20-one in mice. Pharmacol. Biochem. Behav..

[63] Sinnott R.S., Mark G.P., Finn D.A. (2002). Reinforcing effects of the neurosteroid allopregnanolone in rats. Pharmacol. Biochem. Behav..

[64] Purdy R.H., Valenzuela C.F., Janak P.H., Finn D.A., Biggio G., Backstrom T. (2005). Neuroactive steroids and ethanol. Alcohol. Clin. Exp. Res..

[65] Porcu P., Morrow A.L. (2014). Divergent neuroactive steroid responses to stress and ethanol in rat and mouse strains: Relevance for human studies. Psychopharmacology.

[66] Anker J.J., Carroll M.E. (2010). The role of progestins in the behavioral effects of cocaine and other drugs of abuse: Human and animal research. Neurosci. Biobehav. Rev..

[67] VanDoren M.J., Matthews D.B., Janis G.C., Grobin A.C., Devaud L.L., Morrow A.L. (2000). Neuroactive steroid 3α-hydroxy-5α-pregnan-20-one modulates electrophysiological and behavioral actions of ethanol. J. Neurosci..

[68] Porcu P., Sogliano C., Cinus M., Purdy R.H., Biggio G., Concas A. (2003). Nicotine-induced changes in cerebrocortical neuroactive steroids and plasma corticosterone concentrations in the rat. Pharmacol. Biochem. Behav..

[69] Porcu P., Sogliano C., Ibba C., Piredda M., Tocco S., Marra C., Purdy R.H., Biggio G., Concas A. (2004). Failure of gamma-hydroxybutyric acid both to increase neuroactive steroid concentrations in adrenalectomized-orchiectomized rats and to induce tolerance to its steroidogenic effect in intact animals. Brain Res..

[70] Grobin A.C., VanDoren M.J., Porrino L.J., Morrow A.L. (2005). Cortical 3α-hydroxy-5α-pregnan-20-one levels after acute administration of Δ9-tetrahydrocannabinol, cocaine and morphine. Psychopharmacology.

[71] Concas A., Sogliano C., Porcu P., Marra C., Brundu A., Biggio G. (2006). Neurosteroids in nicotine and morphine dependence. Psychopharmacology.

[72] Vallee M., Vitiello S., Bellocchio L., Hebert-Chatelain E., Monlezun S., Martin-Garcia E., Kasanetz F., Baillie G.L., Panin F., Cathala A. (2014). Pregnenolone can protect the brain from cannabis intoxication. Science.

[73] Bolneo E., Chau P.Y.S., Noakes P.G., Bellingham M.C. (2022). Investigating the role of GABA in neural development and disease using mice lacking GAD67 or VGAT genes. Int. J. Mol. Sci..

[74] Sienes Bailo P., Llorente Martín E., Calmarza P., Montolio Breva S., Bravo Gómez A., Pozo Giráldez A., Sánchez-Pascuala Callau J.J., Vaquer Santamaría J.M., Dayaldasani Khialani A., Cerdá Micó C. (2022). The role of oxidative stress in neurodegenerative diseases and potential antioxidant therapies. Adv. Lab. Med..

[75] Melcangi R.C., Garcia-Segura L.M., Ritsner M.S., Weizman A. (2008). Steroid Metabolism in Glial Cells. Neuroactive Steroids in Brain Function, Behavior and Neuropsychiatric Disorders.

[76] Ritsner M.S. (2010). Pregnenolone, dehydroepiandrosterone, and schizophrenia: Alterations and clinical trials. CNS Neurosci. Ther..

[77] Mehdizadeh A., Aali B.S., Hajializadeh Z., Torkzadeh-Mahani S., Esmaeili-Mahani S. (2019). Neurosteroid dehydroepiandrosterone attenuates 6-hydroxydopamine-induced apoptosis in a cell model of Parkinson’s disease. Physiol. Pharm..

[78] Ritsner M.S., Ritsner M.S., Wei man A. (2008). Dehydroepiandrosterone Administration in Treating Medical and Neuropsychiatric Disorders. Neuroactive Steroids in Brain Function, Behavior and Neuropsychiatric Disorders.

[79] Lazaridis I., Charalampopoulos I., Alexaki V.I., Avlonitis N., Pediaditakis I., Efstathopoulos P., Calogeropoulou T., Castanas E., Gravanis A. (2011). Neurosteroid dehydroepiandrosterone interacts with nerve growth factor (NGF) receptors, preventing neuronal apoptosis. PLoS Biol..

[80] Sorwell K.G., Urbanski H.F. (2010). Dehydroepiandrosterone and age-related cognitive decline. Age.

[81] Bassani T.B., Bartolomeo C.S., Oliveira R.B., Ureshino R.P. (2023). Progestogen-mediated neuroprotection in central nervous system disorders. Neuroendocrinology.

[82] Wei J., Xiao G.M. (2013). The neuroprotective effects of progesterone on traumatic brain injury: Current status and future prospects. Acta Pharmacol. Sin..

[83] Stein D.G. (2008). Progesterone exerts neuroprotective effects after brain injury. Brain Res. Rev..

[84] Guennoun R., Labombarda F., Gonzalez Deniselle M.C., Liere P., De Nicola A.F., Schumacher M. (2015). Progesterone and allopregnanolone in the central nervous system: Response to injury and implication for neuroprotection. J. Steroid Biochem. Mol. Biol..

[85] De Nicola A.F., Meyer M., Garay L., Kruse M.S., Schumacher M., Guennoun R., Gonzalez Deniselle M.C. (2022). Progesterone and allopregnanolone neuroprotective effects in the wobbler mouse model of amyotrophic lateral sclerosis. Cell. Mol. Neurobiol..

[86] Gonzalez Deniselle M.C., López-Costa J.J., Saavedra J.P., Pietranera L., Gonzalez S.L., Garay L., Guennoun R., Schumacher M., De Nicola A.F. (2002). Progesterone neuroprotection in the Wobbler mouse, a genetic model of spinal cord motor neuron disease. Neurobiol. Dis..

[87] Theis V., Theiss C. (2015). Progesterone: A universal stimulus for neuronal cells?. Neural Regen. Res..

[88] Legesse D.H., Fan C., Teng J., Zhuang Y., Howard R.J., Noviello C.M., Lindahl E., Hibbs R.E. (2023). Structural insights into opposing actions of neurosteroids on GABA-A receptors. Nat. Commun..

[89] Hernandez G.D., Brinton R.D. (2022). Allopregnanolone: Regenerative therapeutic to restore neurological health. Neurobiol. Stress.

[90] Chu L., Huang Y., Xu Y., Wang L.K., Lu Q. (2021). An LC-APCI(+)-MS/MSbased method for determining the concentration of neurosteroids in the brain of male mice with different gut microbiota. J. Neurosci. Methods.

[91] Djebaili M., Guo Q., Pettus E.H., Hoffman S.W., Stein D.G. (2005). The Neurosteroids progesterone and allopregnanolone reduce cell death, gliosis, and functional deficits after traumatic brain injury in rats. J. Neurotrauma.

[92] Labombarda F., Gonzalez S., Lima A., Roig P., Guennoun R., Schumacher M., De Nicola A.F. (2011). Progesterone attenuates astro- and microgliosis and enhances oligodendrocyte differentiation following spinal cord injury. Exp. Neurol..

[93] Brinton R.D. (2013). Neurosteroids as regenerative agents in the brain: Therapeutic implications. Nat. Rev. Endocrinol..

[94] Adeosun S.O., Hou X., Jiao Y., Zheng B., Henry S., Hill R., He Z., Pani A., Kyle P., Ou X. (2012). Allo-pregnanolone reinstates tyrosine hydroxylase immunoreactive neurons and motor per-formance in an MPTP-lesioned mouse model of Parkinson’s disease. PLoS ONE.

[95] Yang Q.Q., Zhou J.W. (2019). Neuroinflammation in the central nervous system: Symphony of glial cells. Glia.

[96] DiSabato D.J., Quan N., Godbout J.P. (2016). Neuroinflammation: The devil is in the details. J. Neurochem..

[97] Jacobsen B.M., Horwitz K.B. (2012). Progesterone receptors, their isoforms and progesterone regulated transcription. Mol. Cell. Endocrinol..

[98] Aryanpour R., Pasbakhsh P., Zibara K., Namjoo Z., Beigi Boroujeni F., Shahbeigi S., Kashani I.R., Beyer C., Zendehdel A. (2017). Progesterone therapy induces an M1 to M2 switch in microglia phenotype and suppresses NLRP3 inflammasome in a cuprizone-induced demyelination mouse model. Int. Immunopharm..

[99] Lei B., Mace B., Dawson H.N., Warner D.S., Laskowitz D.T., James M.L. (2014). Anti-inflammatory effects of progesterone in lipopolysaccharide-stimulated BV-2 microglia. PLoS ONE.

[100] Prossnitz E.R., Barton M. (2011). The G-protein-coupled estrogen receptor GPER in Health and disease. Nat. Rev. Endocrinol..

[101] Schaufelberger S.A., Rosselli M., Barchiesi F., Gillespie D.G., Jackson E.K., Dubey R.K. (2016). 2-Methoxyestradiol, an endogenous 17β-estradiol metabolite, inhibits microglial proliferation and activation via an estrogen receptor-independent mechanism. Am. J. Physiol. Endocrinol. Metab.

[102] Wang C., Lv X., Jiang C., Davis J.S. (2012). The putative G-protein coupled estrogen receptor agonist G-1 suppresses proliferation of ovarian and breast cancer cells in a GPER-independent manner. Am. J. Tour. Res..

[103] Guan J., Yang B., Fan Y., Zhang J. (2017). GPER agonist G1 attenuates neuroinflammation and dopaminergic neurodegeneration in Parkinson disease. Neuroimmunomodulation.

[104] Alexaki V.I., Fodelianaki G., Neuwirth A., Mund C., Kourgiantaki A., Ieronimaki E., Lyroni K., Troullinaki M., Fujii C., Kanczkowski W. (2018). DHEA inhibits acute microglia-mediated inflammation through activation of the TrkA-Akt1/2-CREB-jmjd3 pathway. Mol. Psychiatr..

[105] Hu X., Fang Z., Wang F., Mei Z., Huang X., Lin Y., Lin Z. (2024). A causal relationship between gut microbiota and subcortical brain structures contributes to the microbiota–gut–brain axis: A Mendelian randomization study. Cereb. Cortex.

[106] Rathour D., Shah S., Khan S., Singh P.K., Srivastava S., Singh S.B., Khatri D.K. (2023). Role of gut microbiota in depression: Understanding molecular pathways, recent research, and future direction. Behav. Brain Res..

[107] Hur H.J., Park H.Y., Kim N. (2022). Gut Microbiota and Depression, Anxiety, and Cognitive Disorders. Sex/Gender-Specific Medicine in the Gastrointestinal Diseases.

[108] Chang L.J., Wei Y., Hashimoto K. (2022). Brain–gut–microbiota axis in depression: A historical overview and future directions. Brain Res. Bull..

[109] Carding S., Verbeke K., Vipond D.T., Corfe B.M., Owen L.J. (2015). Dysbiosis of the gut microbiota in disease. Microb. Ecol. Health Dis..

[110] Liu L.X., Wang H.Y., Rao X.C., Yu Y., Li W.X., Zheng P., Zhao L.B., Zhou C.J., Pu J.C., Yang D.Y. (2021). Comprehensive analysis of the lysine acetylome and succinylome in the hippocampus of gut microbiota-dysbiosis mice. J. Adv. Res..

[111] Sudo N., Chida Y., Aiba Y., Sonoda J., Oyama N., Yu X., Kubo C., Koga Y. (2004). Postnatal Microbial Colonization Programs the Hypothalamic–Pituitary–Adrenal System for Stress Response in Mice. J. Physiol..

[112] Gareau M.G., Wine E., Rodrigues D.M., Cho J.H., Whary M.T., Philpott D.J., MacQueen G., Sherman P.M. (2011). Bacterial Infection Causes Stress-Induced Memory Dysfunction in Mice. Gut.

[113] Heijtz R.D., Wang S., Anuar F., Qian Y., Björkholm B., Samuelsson A., Hibberd M.L., Forssberg H., Pettersson S. (2011). Normal GutMicrobiota Modulates Brain Development and Behavior. Proc. Natl. Acad. Sci. USA.

[114] Neufeld K.M., Kang N., Bienenstock J., Foster J.A. (2011). Reduced Anxiety-like Behavior and Central Neurochemical Change in Germ-Free Mice: Behavior in Germ-Free Mice. Neurogastroenterol. Motil..

[115] Clarke G., Grenham S., Scully P., Fitzgerald P., Moloney R.D., Shanahan F., Dinan T.G., Cryan J.F. (2013). The Microbiome-Gut-Brain Axis during Early Life Regulates the Hippocampal Serotonergic System in a Sex-Dependent Manner. Mol. Psychiatry.

[116] Bercik P., Denou E., Collins J., Jackson W., Lu J., Jury J., Deng Y., Blennerhassett P., Macri J., McCoy K.D. (2011). The Intestinal Microbiota Affect Central Levels of Brain-Derived Neurotropic Factor and Behavior in Mice. Gastroenterology.

[117] Savignac H.M., Kiely B., Dinan T.G., Cryan J.F. (2014). BIfidobacteria Exert Strain-specific Effects on Stress-related Behavior and Physiology in BALB/c Mice. Neurogastroenterol. Motil..

[118] Desbonnet L., Clarke G., Traplin A., O’Sullivan O., Crispie F., Moloney R.D., Cotter P.D., Dinan T.G., Cryan J.F. (2015). Gut Microbiota Depletion from Early Adolescence in Mice: Implications for Brain and Behaviour. Brain Behav. Immun..

[119] Bharwani A., Mian M.F., Surette M.G., Bienenstock J., Forsythe P. (2017). Oral Treatment with Lactobacillus Rhamnosus Attenuates Behavioural Deficits and Immune Changes in Chronic Social Stress. BMC Med..

[120] Cheung S.G., Goldenthal A.R., Uhlemann A.C., Mann J.J., Miller J.M., Sublette M.E. (2019). Systematic Review of Gut Microbiota and Major Depression. Front. Psychiatry.

[121] Caspani G., Kennedy S., Foster J.A., Swann J. (2019). Gut microbial metabolites in depression: Understanding the biochemical mechanisms. Microb. Cell.

[122] Li J., Mei P.C., An N., Fan X.X., Liu Y.Q., Zhu Q.F., Feng Y.Q. (2025). Unraveling the Metabolic and Microbiome Signatures in Fecal Samples of Pregnant Women with Prenatal Depression. Metabolites.

[123] Bai S.J., Bai H.L., Li D.T., Zhong Q., Xie J., Chen J.J. (2022). Gut Microbiota-Related Inflammation Factors as a Potential Biomarker for Diagnosing Major Depressive Disorder. Front. Cell. Infect. Microbiol..

[124] Peirce J.M., Alviña K. (2019). The role of inflammation and the gut microbiome in depression and anxiety. J. Neurosci. Res..

[125] Marchev A.S., Vasileva L.V., Amirova K.M., Savova M.S., Balcheva-Sivenova Z.P., Georgiev M.I. (2021). Metabolomics and health: From nutritional crops and plant-based pharmaceuticals to profiling of human biofluids. Cell. Mol. Life Sci..

[126] Muthubharathi B.C., Gowripriya T., Balamurugan K. (2021). Metabolomics: Small molecules that matter more. Mol. Omics.

[127] An N., Zhang M., Zhu Q.-F., Chen Y.-Y., Deng Y.-L., Liu X.-Y., Zeng Q., Feng Y.-Q. (2024). Metabolomic Analysis Reveals Association between Decreased Ovarian Reserve and In Vitro Fertilization Outcomes. Metabolites.

[128] Xu J., Zhang Q., Zheng J., Yuan B., Feng Y. (2019). Mass spectrometry-based fecal metabolome analysis. TRAC Trends Anal. Chem..

[129] Yuan X.M., Chen B.Q., Duan Z.L., Xia Z.Q., Ding Y., Chen T., Liu H.Z., Wang B.S., Yang B.L., Wang X.Y. (2021). Depression and anxiety in patients with active ulcerative colitis: Crosstalk of gut microbiota, metabolomics and proteomics. Gut Microbes.

[130] Sinha R., Ahn J., Sampson J.N., Shi J., Yu G., Xiong Q., Hayes R.B., Goedert J.J. (2016). Fecal Microbiota, Fecal Metabolome, and Colorectal Cancer Interrelations. PLoS ONE.

[131] Zhou L., Ni Z.X., Yu J., Cheng W., Cai Z.L., Yu C.Q. (2020). Correlation Between Fecal Metabolomics and Gut Microbiota in Obesity and Polycystic Ovary Syndrome. Front. Endocrinol..

[132] Montagnani M., Bottalico L., Potenza M.A., Charitos I.A., Topi S., Colella M., Santacroce L. (2023). The Crosstalk between Gut Microbiota and Nervous System: A Bidirectional Interaction between Microorganisms and Metabolome. Int. J. Mol. Sci..

[133] Liu N., Sun S., Wang P., Sun Y., Hu Q., Wang X. (2021). The Mechanism of Secretion and Metabolism of Gut-Derived 5-Hydroxytryptamine. Int. J. Mol. Sci..

[134] Gao K., Mu C., Farzi A., Zhu W. (2020). Tryptophan Metabolism: A Link Between the Gut Microbiota and Brain. Adv. Nutr..

[135] Dicks L.M.T. (2022). Gut Bacteria and Neurotransmitters. Microorganisms.

[136] Den Besten G., Van Eunen K., Groen A.K., Venema K., Reijngoud D.J., Bakker B.M. (2013). The role of short-chain fatty acids in the interplay between diet, gut microbiota, and host energy metabolism. J. Lipid Res..

[137] Wong J.M.W., De Souza R., Kendall C.W.C., Emam A., Jenkins D.J.A. (2006). Colonic Health: Fermentation and Short Chain Fatty Acids. J. Clin. Gastroenterol..

[138] Louis P., Hold G.L., Flint H.J. (2014). The gut microbiota, bacterial metabolites and colorectal cancer. Nat. Rev. Microbiol..

[139] Koh A., De Vadder F., Kovatcheva-Datchary P., Bäckhed F. (2016). From dietary fiber to host physiology: Short-chain fatty acids as key bacterial metabolites. Cell.

[140] Anand S., Kaur H., Mande S.S. (2016). Comparative in silico analysis of butyrate production pathways in gut commensals and pathogens. Front. Microbiol..

[141] Vital M., Howe A.C., Tiedje J.M. (2014). Revealing the bacterial butyrate synthesis pathways by analyzing (meta)genomic data. MBio.

[142] Oldendorf W.H. (1973). Carrier-Mediated Blood-Brain Barrier Transport of Short-Chain Monocarboxylic Organic Acids. www.physiology.org/journal/ajplegacy.

[143] Bourassa M.W., Alim I., Bultman S.J., Ratan R.R. (2016). Butyrate, neuroepigenetics and the gut microbiome: Can a high fiber diet improve brain health?. Neurosci. Lett..

[144] van de Wouw M., Boehme M., Lyte J.M., Wiley N., Strain C., O’Sullivan O., Clarke G., Stanton C., Dinan T.G., Cryan J.F. (2018). Short-chain fatty acids: Microbial metabolites that alleviate stress-induced brain–gut axis alterations. J. Physiol..

[145] Sherwin E., Rea K., Dinan T.G., Cryan J.F. (2016). A gut (microbiome) feeling about the brain. Curr. Opin. Gastroenterol..

[146] Parker A., Fonseca S., Carding S.R. (2020). Gut microbes and metabolites as modulators of blood-brain barrier integrity and brain health. Gut Microbes.

[147] Liddle R.A. (2019). Neuropods. Cell. Mol. Gastroenterol. Hepatol..

[148] Silva Y.P., Bernardi A., Frozza R.L. (2020). The Role of Short-Chain Fatty Acids from Gut Microbiota in Gut-Brain Communication. Front. Endocrinol..

[149] Takata F., Nakagawa S., Matsumoto J., Dohgu S. (2021). Blood-Brain Barrier Dysfunction Amplifies the Development of Neuroinflammation: Understanding of Cellular Events in Brain Microvascular Endothelial Cells for Prevention and Treatment of BBB Dysfunction. Front. Cell. Neurosci..

[150] Braniste V., Al-Asmakh M., Kowal C., Anuar F., Abbaspour A., Tóth M., Korecka A., Bakocevic N., Ng L.G., Kundu P. (2014). The gut microbiota influences blood-brain barrier permeability in mice. Sci. Transl. Med..

[151] Hoyles L., Snelling T., Umlai U.-K., Nicholson J.K., Carding S.R., Glen R.C., McArthur S. (2018). Microbiome–host systems interactions: Protective effects of propionate upon the blood–brain barrier. Microbiome.

[152] Fock E., Parnova R. (2023). Mechanisms of Blood–Brain Barrier Protection by Microbiota-Derived Short-Chain Fatty Acids. Cells.

[153] Cheng J., Hu H., Ju Y., Liu J., Wang M., Liu B., Zhang Y. (2024). Gut microbiota-derived short-chain fatty acids and depression: Deep insight into biological mechanisms and potential applications. Gen. Psychiatry.

[154] Chen T., Noto D., Hoshino Y., Mizuno M., Miyake S. (2019). Butyrate suppresses demyelination and enhances remyelination. J. Neuroinflamm..

[155] Dong Y., Cui C. (2022). The role of short-chain fatty acids in central nervous system diseases. Mol. Cell. Biochem..

[156] Swer N.M., Venkidesh B.S., Murali T.S., Mumbrekar K.D. (2023). Gut microbiota-derived metabolites and their importance in neurological disorders. Mol. Biol. Rep..

[157] Singh V., Lee G., Son H., Koh H., Kim E.S., Unno T., Shin J.-H. (2023). Butyrate producers, ‘The Sentinel of Gut’: Their intestinal significance with and beyond butyrate, and prospective use as microbial therapeutics. Front. Microbiol..

[158] Vargas-Caraveo A., Sayd A., Robledo-Montaña J., Caso J.R., Madrigal J.L.M., García-Bueno B., Leza J.C. (2020). Toll-like receptor 4 agonist and antagonist lipopolysaccharides modify innate immune response in rat brain circumventricular organs. J. Neuroinflamm..

[159] Muzio L., Viotti A., Martino G. (2021). Microglia in Neuroinflammation and Neurodegeneration: From Understanding to Therapy. Front. Neurosci..

[160] Wenzel T.J., Gates E.J., Ranger A.L., Klegeris A. (2020). Short-chain fatty acids (SCFAs) alone or in combination regulate select immune functions of microglia-like cells. Mol. Cell. Neurosci..

[161] Wang P., Zhang Y., Gong Y., Yang R., Chen Z., Hu W., Wu Y., Gao M., Xu X., Qin Y. (2018). Sodium butyrate triggers a functional elongation of microglial process via Akt-small RhoGTPase activation and HDACs inhibition. Neurobiol. Dis..

[162] Rothhammer V., Mascanfroni I.D., Bunse L., Takenaka M.C., Kenison J.E., Mayo L., Chao C.-C., Patel B., Yan R., Blain M. (2016). Type I interferons and microbial metabolites of tryptophan modulate astrocyte activity and central nervous system inflammation via the aryl hydrocarbon receptor. Nat. Med..

[163] Hoban A.E., Stilling R.M., Ryan F.J., Shanahan F., Dinan T.G., Claesson M.J., Clarke G., Cryan J.F. (2016). Regulation of prefrontal cortex myelination by the microbiota. Transl. Psychiatry.

[164] Kenny D.J., Plichta D.R., Shungin D., Koppel N., Hall A.B., Fu B., Vasan R.S., Shaw S.Y., Vlamakis H., Balskus E.P. (2020). Cholesterol Metabolism by Uncultured Human Gut Bacteria Influences Host Cholesterol Level. Cell Host Microbe.

[165] Midtvedt T., Lingaas E., Carlstedt-Duke B., Höverstad T., Midtvedt A.C., Saxerholt H., Steinbakk M., Norin K.E. (1990). Intestinal microbial conversion of cholesterol to coprostanol in man. APMIS.

[166] Skolnick S. (2024). Prospecting for para-endogenous anxiolytics in the human microbiome: Some promising pathways. Pharmacol. Biochem. Behav..

[167] Osborne L.M., Gispen F., Sanyal A., Yenokyan G., Meilman S., Payne J.L. (2017). Lower allopregnanolone during pregnancy predicts postpartum depression: An exploratory study. Psychoneuroendocrinology.

[168] Boonstra E., de Kleijn R., Colzato L.S., Alkemade A., Forstmann B.U., Nieuwenhuis S. (2015). Neurotransmitters as food supplements: The effects of GABA on brain and behavior. Front. Psychol..

[169] Hepsomali P., Groeger J.A., Nishihira J., Scholey A. (2020). Effects of oral gamma-aminobutyric acid (GABA) administration on stress and sleep in humans: A systematic review. Front. Neurosci..

[170] Louveau A., Smirnov I., Keyes T.J., Eccles J.D., Rouhani S.J., Peske J.D., Derecki N.C., Castle D., Mandell J.W., Lee K.S. (2015). Structural and functional features of central nervous system lymphatic vessels. Nature.

[171] Kazemi A., Soltani S., Ghorabi S., Keshtkar A., Daneshzad E., Nasri F., Mazloomi S.M. (2020). Effect of probiotic and synbiotic supplementation on inflammatory markers in health and disease status: A systematic review and meta-analysis of clinical trials. Clin. Nutr..

[172] Belelli D., Lambert J.J., Wan M.L.Y., Monteiro A.R., Nutt D.J., Swinny J.D. (2025). From bugs to brain: Unravelling the GABA signalling networks in the brain-gut-microbiome axis. Brain.

[173] Salminen S., Collado M.C., Endo A., Hill C., Lebeer S., Quigley E.M.M., Sanders M.E., Shamir R., Swann J.R., Szajewska H. (2022). The International Scientific Association of Probiotics and Prebiotics (ISAPP) consensus statement on the definition and scope of postbiotics. Nat. Rev. Gastroenterol. Hepatol..

[174] Braga J.D., Thongngam M., Kumrungsee T. (2024). Gamma-aminobutyric acid as a potential postbiotic mediator in the gut-brain axis. NPJ Sci. Food.

[175] Messaoudi M., Violle N., Bisson J.F., Desor D., Javelot H., Rougeot C. (2011). Beneficial psychological effects of a probiotic formulation (Lactobacillus helveticus R0052 and Bifidobacterium longum R0175) in healthy human volunteers. Gut Microbes.

[176] Firth J., Marx W., Dash S., Carney R., Teasdale S.B., Solmi M., Stubbs B., Schuch F.B., Carvalho A.F., Jacka F. (2019). The effects of dietary improvement on symptoms of depression and anxiety: A meta-analysis of randomized controlled trials. Psychosom. Med..

[177] Bayes J., Schloss J., Sibbritt D. (2022). The effect of a Mediterranean diet on the symptoms of depression in young males (the “AMMEND: A Mediterranean diet in MEN with depression” study): A randomized controlled trial. Am. J. Clin. Nutr..

[178] Leclercq S., de Timary P., Delzenne N.M., Starkel P. (2017). The link between inflammation, bugs, the intestine and the brain in alcohol dependence. Transl. Psychiatry.

[179] Bagga D., Reichert J.L., Koschutnig K., Aigner C.S., Holzer P., Koskinen K., Moissl-Eichinger C., Schöpf V. (2018). Probiotics drive gut microbiome triggering emotional brain signatures. Gut Microbes.

[180] Barros-Santos T., Silva K.S.O., Libarino-Santos M., Cata-Preta E.G., Reis H.S., Tamura E.K., de Oliveira-Lima A.J., Berro L.F., Uetanabaro A.P.T., Marinho E.A.V. (2020). Effects of chronic treatment with new strains of Lactobacillus plantarum on cognitive, anxiety- and depressive-like behaviors in male mice. PLoS ONE.

[181] Munawar N., Ahmad A., Anwar M.A., Muhammad K. (2022). Modulation of gut microbial diversity through non-pharmaceutical approaches to treat schizophrenia. Int. J. Mol. Sci..

[182] Pokusaeva K., Johnson C., Luk B., Uribe G., Fu Y., Oezguen N., Matsunami R.K., Lugo M., Major A., Mori-Akiyama Y. (2017). GABA-producing Bifidobacterium dentium modulates visceral sensitivity in the intestine. Neurogastroenterol. Motil..

[183] Bagheri S., Heydari A., Alinaghipour A., Salami M. (2019). Effect of probiotic supplementation on seizure activity and cognitive performance in PTZ-induced chemical kindling. Epilepsy Behav..

[184] Angoa-Perez M., Kuhn D.M. (2021). Evidence for modulation of substance use disorders by the gut microbiome: Hidden in plain sight. Pharmacol. Rev..

[185] Roberfroid M.B. (2005). Introducing inulin-type fructans. Br. J. Nutr..

[186] Suligoj T., Vigsnaes L.K., Van den Abbeele P., Apostolou A., Karalis K., Savva G.M., McConnell B., Juge N. (2020). Effects of human milk oligosaccharides on the adult gut microbiota and barrier function. Nutrients.

[187] Barrett E., Ross R.P., O’Toole P.W., Fitzgerald G.F., Stanton C. (2012). γ-Aminobutyric acid production by culturable bacteria from the human intestine. J. Appl. Microbiol..

[188] Duranti S., Ruiz L., Lugli G.A., Tames H., Milani C., Mancabelli L., Mancino W., Longhi G., Carnevali L., Sgoifo A. (2020). Bifidobacterium adolescentis as a key member of the human gut microbiota in the production of GABA. Sci. Rep..

[189] Briggs J.A., Grondin J.M., Brumer H. (2021). Communal living: Glycan utilization by the human gut microbiota. Environ. Microbiol..

[190] Bajic D., Wiens F., Wintergerst E., Deyaert S., Baudot A., Van den Abbeele P. (2023). HMOs exert marked bifidogenic effects on children’s gut microbiota ex vivo, due to age-related Bifidobacterium species composition. Nutrients.

[191] Fan S., Zhang Z., Zhao Y., Daglia M., Zhang J., Zhu Y., Bai J., Zhu L., Xiao X. (2023). Recent advances in targeted manipulation of the gut microbiome by prebiotics: From taxonomic composition to metabolic function. Curr. Opin. Food Sci..

[192] Jackson P.P.J., Wijeyesekera A., Rastall R.A. (2023). Oligofructose alone and in combination with 2′fucosyllactose induces physiologically relevant changes in gamma-aminobutyric acid and organic acid production compared to sole 2′fucosyllactose supplementation: An in vitro study. FEMS Microbiol. Ecol..

[193] Pferschy-Wenzig E.M., Pausan M.R., Ardjomand-Woelkart K., Röck S., Ammar R.M., Kelber O., Moissl-Eichinger C., Bauer R. (2022). Medicinal plants and their impact on the gut microbiome in mental health: A systematic review. Nutrients.

[194] Wan M.L.Y., Co V.A., El-Nezami H. (2021). Dietary polyphenol impact on gut health and microbiota. Crit. Rev. Food Sci. Nutr..

[195] Scott K.P., Grimaldi R., Cunningham M., Sarbini S.R., Wijeyesekera A., Tang M.L.K., Lee J.C., Yau Y.F., Ansell J., Theis S. (2020). Developments in understanding and applying prebiotics in research and practice-an ISAPP conference paper. J. Appl. Microbiol..

[196] Rodriguez-Daza M.C., Pulido-Mateos E.C., Lupien-Meilleur J., Guyonnet D., Desjardins Y., Roy D. (2021). Polyphenol-mediated gut microbiota modulation: Toward prebiotics and further. Front. Nutr..

[197] Jackson P.P.J., Wijeyesekera A., Rastall R.A. (2022). Determining the metabolic fate of human milk oligosaccharides: It may just be more complex than you think?. Gut Microbiome.

[198] Culp E.J., Goodman A.L. (2023). Cross-feeding in the gut microbiome: Ecology and mechanisms. Cell Host Microbe.

[199] Jolivel V., Brun S., Binamé F., Benyounes J., Taleb O., Bagnard D., De Sèze J., Patte-Mensah C., Mensah-Nyagan A.G. (2021). Microglial cell morphology and phagocytic activity are critically regulated by the neurosteroid allopregnanolone: A possible role in neuroprotection. Cells.

[200] Lucchi C., Codeluppi A., Filaferro M., Vitale G., Rustichelli C., Avallone R., Mandrioli J., Biagini G. (2023). Human microglia synthesize NS to cope with rotenone-induced oxidative stress. Antioxidants.

[201] Bali N., Arimoto J.M., Morgan T.E., Finch C.E. (2013). Progesterone antagonism of neurite outgrowth depends on microglial activation via Pgrmc1/S2R. Endocrinology.

[202] Cutler S.M., Cekic M., Miller D.M., Wali B., VanLandingham J.W., Stein D.G. (2007). Progesterone improves acute recovery after traumatic brain injury in the aged rat. J. Neurotrauma.

[203] Hua F., Wang J., Ishrat T., Wei W., Atif F., Sayeed I., Stein D.G. (2011). Genomic profile of toll-like receptor pathways in traumatically brain-injured mice: Effect of exogenous progesterone. J. Neuroinflamm..

[204] Garay L., Gonzalez Deniselle M.C., Lima A., Roig P., De Nicola A.F. (2007). Effects of progesterone in the spinal cord of a mouse model of multiple sclerosis. J. Steroid Biochem. Mol. Biol..

[205] Gold S.M., Voskuhl R.R. (2009). Estrogen and Testosterone therapies in multiple sclerosis. Prog. Brain Res..

[206] Aggelakopoulou M., Kourepini E., Paschalidis N., Simoes D.C.M., Kalavrizioti D., Dimisianos N., Papathanasopoulos P., Mouzaki A., Panoutsakopoulou V. (2016). Erβ-dependent direct suppression of human and murine Th17 cells and treatment of established central nervous system autoimmunity by a neurosteroid. J. Immunol..

[207] Boghozian R., McKenzie B.A., Saito L.B., Mehta N.B., William G., Lu J., Baker G.B., Noorbakhsh F., Power C. (2017). Suppressed oligodendrocyte steroidogenesis in multiple sclerosis: Implications for regulation of neuroinflammation. Glia.

[208] Saijo K., Collier J.G., Li A.C., Katzenellenbogen J.A., Glass C.K. (2011). An ADIOL-ERβ-CtBP transrepression pathway negatively regulates microglia-mediated inflammation. Cell.

[209] Servi R., Akkoç R.F., Aksu F., Servi S. (2025). Therapeutic potential of enzymes, neurosteroids, and synthetic steroids in neurodegenerative disorders: A critical review. J. Steroid Biochem. Mol. Biol..

[210] Kanes S., Colquhoun H., Gunduz-Bruce H., Raines S., Arnold R., Schacterle A., Doherty J., Epperson C.N., Deligiannidis K.M., Riesenberg R. (2017). Brexanolone (SAGE-547 injection) in post-partum depression: A randomised controlled trial. Lancet.

[211] Epperson C.N., Rubinow D.R., Meltzer-Brody S., Deligiannidis K.M., Riesenberg R., Krystal A.D., Bankole K., Huang M.-Y., Li H., Brown C. (2023). Effect of brexanolone on depressive symptoms, anxiety, and insomnia in women with postpartum depression: Pooled analyses from 3 double-blind, randomized, placebo-controlled clinical trials in the HUMMINGBIRD clinical program. J. Affect. Disord..

[212] Deligiannidis K.M., Citrome L., Huang M.Y., Acaster S., Fridman M., Bonthapally V., Lasser R., Kanes S.J. (2023). Effect of zuranolone on concurrent anxiety and insomnia symptoms in women with postpartum depression. J. Clin. Psychiatry.

[213] Gunduz-Bruce H., Silber C., Kaul I., Rothschild A.J., Riesenberg R., Sankoh A.J., Li H., Lasser R., Zorumski C.F., Rubinow D.R. (2019). Trial of SAGE-217 in patients with major depressive disorder. N. Engl. J. Med..

[214] Bullock A., Gunduz-Bruce H., Zammit G.K., Qin M., Li H., Sankoh A.J., Silber C., Kanes S.J., Jonas J., Doherty J. (2022). A phase 1 double-blind, placebo-controlled study of zuranolone (SAGE-217) in a phase advance model of insomnia in healthy adults. Hum. Psychopharmacol..

[215] Bäckström T., Ekberg K., Hirschberg A.L., Bixo M., Epperson C.N., Briggs P., Panay N., O’bRien S. (2021). A randomized, double-blind study on efficacy and safety of sepranolone in premenstrual dysphoric disorder. Psychoneuroendocrinology.

[216] Dichtel L.E., Nyer M., Dording C., Fisher L.B., Cusin C., Shapero B.G., Pedrelli P., Kimball A.S., Rao E.M., Mischoulon D. (2020). Effects of open-label, adjunctive ganaxolone on persistent depression despite adequate antidepressant treatment in postmenopausal women: A pilot study. J. Clin. Psychiatry.

[217] Vaitkevicius H., Ramsay R.E., Swisher C.B., Husain A.M., Aimetti A., Gasior M. (2022). Intravenous ganaxolone for the treatment of refractory status epilepticus: Results from an open-label, dose-finding, phase 2 trial. Epilepsia.

[218] Sullivan J., Gunning B., Zafar M., Guerrini R., Gecz J., Kolc K.L., Zhao Y., Gasior M., Aimetti A.A., Samanta D. (2023). Phase 2, placebo-controlled clinical study of oral ganaxolone in PCDH19-clustering epilepsy. Epilepsy Res..

[219] Yawno T., Mahen M., Li J., Fahey M.C., Jenkin G., Miller S.L. (2017). The beneficial effects of melatonin administration following hypoxia-ischemia in preterm fetal sheep. Front. Cell. Neurosci..

[220] Mouihate A., Kalakh S. (2019). Ganaxolone enhances microglial clearance activity and promotes remyelination in focal demyelination in the corpus callosum of ovariectomized rats. CNS Neurosci. Ther..

[221] Tateiwa H., Evers A.S. (2024). Neurosteroids and their potential as a safer class of general anesthetics. J. Anesth..

[222] Reddy D.S. (2009). The role of neurosteroids in the pathophysiology and treatment of catamenial epilepsy. Epilepsy Res..

[223] Nohria V., Giller E. (2007). Ganaxolone. Neurotherapeutics.

[224] Balan I., Boero G., Chéry S.L., McFarland M.H., Lopez A.G., Morrow A.L. (2024). Neuroactive Steroids, Toll-like Receptors, and Neuroimmune Regulation: Insights into Their Impact on Neuropsychiatric Disorders. Life.

[225] Singhal M., Modi N., Bansal L., Abraham J., Mehta I., Ravi A. (2024). The Emerging Role of NS: Novel Drugs Brexanalone, Sepranolone, Zuranolone, and Ganaxolone in Mood and Neurological Disorders. Cureus.

[226] Chatterjee P., Dassama L.M.K. (2024). Unveiling of a messenger: Gut microbes make a neuroactive signal. Cell.

